# MAPK Cascade Signaling Is Involved in α-MMC Induced Growth Inhibition of Multiple Myeloma MM.1S Cells via G2 Arrest and Mitochondrial-Pathway-Dependent Apoptosis In Vitro

**DOI:** 10.3390/ph16010124

**Published:** 2023-01-13

**Authors:** Zi-Wei Cai, Ting Ye, Pei-Wen Jiang, Yu-Jiao Liao, Lin Wang, Qing-Liang Zhang, Wen-Qian Du, Min Huang, Ping Yang, Min-Hui Li

**Affiliations:** 1 School of Basic Medicine, Chengdu Medical College, Xindu 610500, China; 2 Department of Laboratory Medicine, Jin Tang First People’s Hospital, Jintang 610400, China; 3 School of Pharmacy, Chengdu Medical College, Xindu 610500, China; 4 School of Bioscience and Technology, Chengdu Medical College, Xindu 610500, China; 5 Center of Scientific Research and Experiment, Chengdu Medical College, Xindu 610500, China

**Keywords:** α-momorcharin, multiple myeloma, mitochondrial-pathway-dependent apoptosis, MAPK cascade signaling, G2 arrest

## Abstract

Multiple myeloma is a hematological malignancy characterized by the unrestricted proliferation of plasma cells that secrete monoclonal immunoglobulins in the bone marrow. Alpha-momorcharin (α-MMC) is a type I ribosome-inactivating protein extracted from the seeds of the edible plant *Momordica charantia* L., which has a variety of biological activities. This study aimed to investigate the inhibitory effect of α-MMC on the proliferation of multiple myeloma MM.1S cells and the molecular mechanism of MM.1S cell death induced through the activation of cell signal transduction pathways. The cell counting kit-8 (CCK-8) assay was used to determine the inhibitory effect of α-MMC on the proliferation of MM.1S cells and its toxic effect on normal human peripheral blood mononuclear cells (PBMCs). The effect of α-MMC on the MM.1S cells’ morphology was observed via inverted microscope imaging. The effects of α-MMC on the MM.1S cell cycle, mitochondrial membrane potential (MMP), and apoptosis were explored using propidium iodide, JC-1, annexin V- fluorescein isothiocyanate/propidium iodide fluorescence staining, and flow cytometry (FCM) analysis. Western blot was used to detect the expressions levels of apoptosis-related proteins and MAPK-signaling-pathway-related proteins in MM.1S cells induced by α-MMC. The results of the CCK-8 showed that in the concentration range of no significant toxicity to PBMCs, α-MMC inhibited the proliferation of MM.1S cells in a time-dependent and concentration-dependent manner, and the IC_50_ value was 13.04 and 7.518 μg/mL for 24 and 48 h, respectively. Through inverted microscope imaging, it was observed that α-MMC induced a typical apoptotic morphology in MM.1S cells. The results of the FCM detection and analysis showed that α-MMC could arrest the MM.1S cells cycle at the G2 phase, decrease the MMP, and induce cell apoptosis. Western blot analysis found that α-MMC upregulated the expression levels of Bax, Bid, cleaved caspase-3, and cleaved PARP, and downregulated the expression levels of Mcl-1. At the same time, α-MMC decreased the expression levels of p-c-Raf, p-MEK1/2, p-ERK1/2, p-MSK1, and p-P90RSK, and increased the expression levels of p-p38, p-SPAK/JNK, p-c-Jun, and p-ATF2. The above results suggest that α-MMC can inhibit the proliferation of multiple myeloma MM.1S cells. MAPK cascade signaling is involved in the growth inhibition effect of α-MMC on MM.1S cells via cycle arrest and mitochondrial-pathway-dependent apoptosis.

## 1. Introduction

Multiple myeloma (MM) is the second most common hematological malignancy after lymphoma, accounting for approximately 10% of all hematological tumors. It can cause damage to related organs and tissues and eventually lead to lytic bone lesions, anemia, renal failure, hypercalcemia, and the so-called “CRAB” symptoms [1]. Therapies based on proteasome inhibitors (PIs), immunomodulatory drugs (IMiDs), and monoclonal antibodies (MoAbs) have been widely used to treat MM patients, but MM remains an incurable disease [2,3,4]. The main reason for the treatment failure of MM patients is that it is difficult to eradicate the minimal residual lesions, and the effects of drug treatment will gradually weaken with the recurrence of the disease, eventually producing drug resistance [5]. Previous studies have reported that the median overall survival of patients with disease relapse and drug resistance after treatment with PIs and IMiDs is only 13 months [6]. These patients usually have higher rates of proliferation and drug resistance, which indicates that clinical medicine has an urgent requirement for new and effective anti-MM drugs. The active substrate isolated from edible plants can inhibit the carcinogenic process by regulating the cell homeostasis and cell death mechanisms, which has great potential in the fight against cancer [7].

Ribosome-inactivating proteins (RIPs) are high-efficiency protein toxins widely distributed in higher plants, with various biological functions [8]. It has been reported that the biological functions of RIPs largely depend on their RNA N-glycosidase activity. RIPs act on ribosomal RNA (rRNA) to remove specific adenine residues under RNA N-glycosidase activity, destroy the ribosome structure, and block the binding of ribosome and protein synthesis elongation factor 2 (eEF2), thereby irreversibly inhibiting protein biosynthesis [9]. Researchers believe that RIPs have potential as antitumor drugs. As protein toxins, due to their large-size effect, RIPs can effectively remain inside cells and avoid drug efflux mediated by multidrug resistance transporters, allowing RIPs to inhibit protein synthesis and kill tumor cells at low concentrations, which is incomparable to other small anticancer drugs [10,11].

Alpha-momorcharin (α-MMC) is a type I RIP isolated from the seeds of the edible plant *Momordica charantia L.*, commonly known as bitter melon [12]. α-MMC has a wide range of biological activities, such as antiviral, antibacterial, and immunomodulatory activities [13]. A few studies have reported the antitumor activity of α-MMC and have found that α-MMC can inhibit the proliferation of different tumor cells, including breast cancer, colon cancer, and non-small-cell lung cancer, but the toxicity to normal cells is low [14,15,16,17]. However, there are no studies that have elucidated the biological effects of α-MMC on MM. Meanwhile, the antitumor activity of α-MMC mediated by the MAPK signaling pathway has not been reported in depth. Reviewing the activity mechanism of α-MMC, it was found that the MAPK signaling pathway is involved in regulating the immunosuppressive activity of α-MMC; α-MMC regulated the secretion of cytokines in monocytes/macrophages via the MAPK signaling pathway [18,19]. Based on the above information, this study took MM.1S cells as a representative subject to explore the molecular mechanism by which α-MMC activates MAPK cascade signaling to exert anti-MM activity. The results provide new insights into the antitumor mechanism of α-MMC, which will be helpful for the potential application of α-MMC as a natural anti-MM drug.

## 2. Results

### 2.1. α-MMC Inhibited the Proliferation of MM.1S Cells

**Figure 1:**
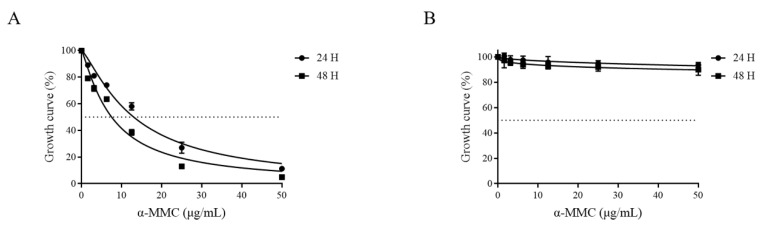
Growth inhibitory effect of α-MMC on MM.1S cells and PBMCs. The cells were treated with α-MMC for 24 and 48 h, and then the cell viability was detected using the CCK-8 method. The growth curves of the MM.1S cells (**A**) and PBMCs (**B**) were drawn using GraphPad Prism 9.0 software.

The results of the cell counting kit-8 (CCK-8) assay showed that α-MMC inhibited the proliferation of MM.1S cells in a time-dependent and concentration-dependent manner, and the half-maximal inhibitory concentration (IC_50_) value was 13.04 and 7.518 μg/mL for 24 and 48 h, respectively. At the same time, within an effective concentration range, α-MMC had no obvious toxicity to peripheral blood mononuclear cells (PBMCs). Compared with the 0 μg/mL α-MMC group, the cell viability of PBMCs treated with 50 μg/mL α-MMC was 93.46 ± 2.10% and 89.89 ± 4.46% for 24 and 48 h (Figure 1).

### 2.2. α-MMC Induced Morphological Changes in MM.1S Cells

**Figure 2:**
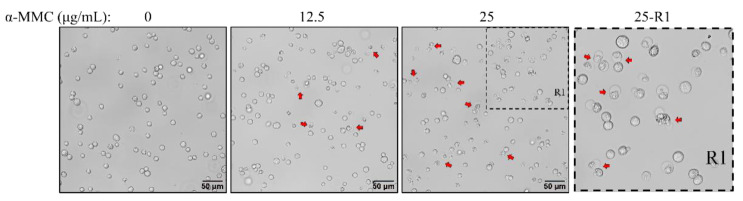
Effect of α-MMC on the morphology of MM.1S cells. After treatment with α-MMC for 24 h, the morphological changes in the MM.1S cells were observed under an inverted microscope. Scale bar 50 μm (20× magnification). The red arrows indicate cells with apoptotic changes in morphology.

After being treated with different concentrations of α-MMC for 24 h, morphological changes in the MM.1S cells were observed using an inverted microscope. The results showed that α-MMC induced a typical apoptotic cell morphology. Compared with the 0 μg/mL α-MMC group, in the 12.5 and 25 μg/mL α-MMC groups, the number of MM.1S cells was reduced, the cell volume was shrunk, and the density of cytoplasm was increased (Figure 2).

### 2.3. α-MMC Induced MM.1S Cell Cycle Arrest at the G2 Phase

**Figure 3:**
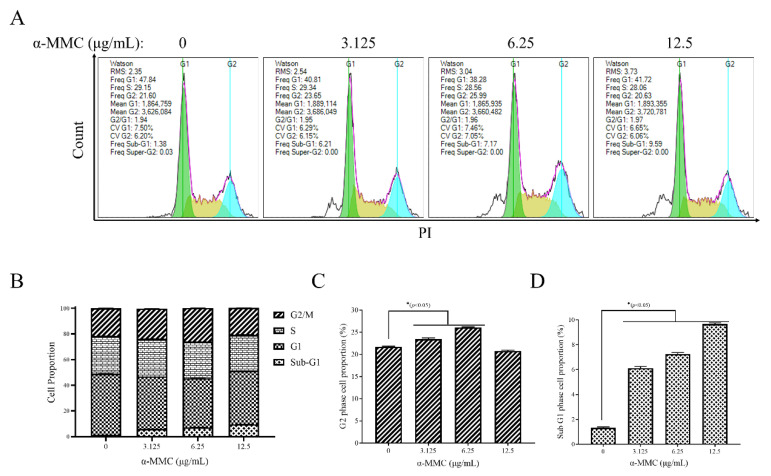
The cell cycle arrest effect of α-MMC on MM.1S cells. After being treated with α-MMC for 12 h, 10,000 counts of PI-stained MM.1S cells were collected via FCM to analyze the cell cycle distribution (**A**); GraphPad Prism 9.0 software was used to analyze the proportions of cells in the different phases of the cell cycle (**B**); the cell proportion in the G2 phase (**C**); the cell proportion in the sub-G1 phase (**D**). In the experimental group (3.125, 6.25, 12.5 μg/mL α-MMC group) comparisons with the control group (0 μg/mL α-MMC group), * *p* < 0.05 was considered to be statistically significant.

To elucidate the mechanism of antiproliferation activity of α-MMC, the cell cycle distribution of MM.1S cells was analyzed using propidium iodide (PI) nucleic acid staining and flow cytometry (FCM) after treatment with α-MMC for 12 h. It was found that the proportion of MM.1S cells in the G2 phase was 21.70 ± 0.14%, 23.46 ± 0.27%, 26.08 ± 0.13%, and 20.77 ± 0.20% after treatment with 0, 3.125, 6.25, and 12.5 μg/mL α-MMC, respectively (Figure 3A,C). In addition, with the increase in the α-MMC concentration, the proportion of MM.1S cells in the sub-G1 phase increased significantly. Compared with the 0 μg/mL α-MMC group, the proportion of MM.1S cells in the sub-G1 phase in the 12.5 μg/mL α-MMC group increased from 1.33 ± 0.07% to 9.66 ± 0.10%, and the difference was statistically significant (Figure 3A,D). These results suggest that α-MMC could arrest the MM.1S cell cycle and, subsequently, induce cell apoptosis. 

### 2.4. α-MMC Induced MM.1S Cell Apoptosis via the Mitochondrial Pathway 

**Figure 4:**
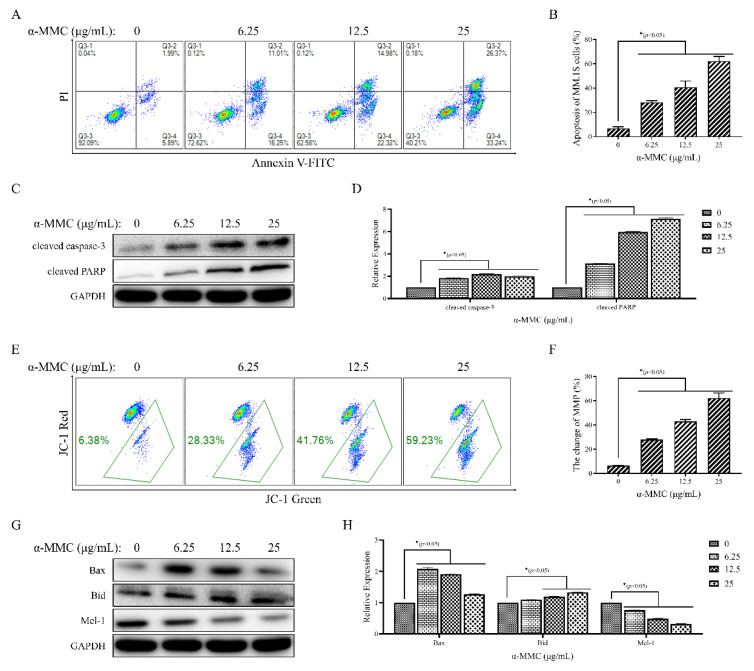
Effect of α-MMC on apoptosis of MM.1S cells. After being treated with α-MMC for 24 h, 5000 counts of MM.1S cells stained with annexin V-FITC/PI (**A**) and JC-1 fluorescence probe (**E**) were collected using FCM, and then the effects of α-MMC on MM.1S cells’ apoptosis and MMP were analyzed. Western blot was used to detect the expression levels of apoptosis-related proteins at the protein level (**C**,**G**). GraphPad Prism 9.0 software was used to draw a histogram to reflect the percentage of apoptosis, the change rate of MMP, and the relative expression levels of proteins (**B**,**D**,**F**,**H**). In the experimental group (6.25, 12.5, 25 μg/mL α-MMC group) comparisons with the control group (0 μg/mL α-MMC group), * *p* < 0.05 was considered to be statistically significant.

To further confirm the apoptosis-inducing effect of α-MMC on MM.1S cells, MM.1S cells stained with annexin V- fluorescein isothiocyanate (FITC) and PI were detected using FCM after being treated with α-MMC for 24 h. The staining results showed that the apoptosis rate of the MM.1S cells increased significantly. The apoptosis rate of the MM.1S cells treated with 0, 6.25, 12.5, and 25 μg/mL α-MMC was 6.99 ± 1.26%, 28.33 ± 1.51%, 40.86 ± 5.03%, and 62.33 ± 3.85%, respectively (Figure 4A,B). Western blot was used to detect the expression levels of apoptosis-related proteins, the results showed that α-MMC significantly increased the expression levels of cleaved caspase-3 and cleaved PARP (Figure 4C,D). These results confirm that α-MMC inhibited the proliferation of MM.1S cells by inducing apoptosis. 

The mitochondrial pathway is a well-known and important pathway involved in programmed cell death. To investigate whether the mitochondrial pathway is involved in the process of α-MMC-induced MM.1S cell death, JC-1 staining was performed on MM.1S cells after their treatment with α-MMC for 24 h. The FCM-detected results showed that the mitochondrial membrane potential (MMP) of the MM.1S cells decreased by 6.44 ± 0.08%, 28 ± 0.47%, 42.89 ± 1.59%, and 62.23 ± 4.24% under treatment with 0, 6.25, 12.5, and 25 μg/mL α-MMC, respectively (Figure 4E,F). Subsequently, the expression levels of Bcl-2-family proteins in the mitochondrial pathway was detected using Western blot, and the results found that α-MMC regulated the expression levels of pro-apoptotic proteins Bax and Bid and downregulated the expression levels of anti-apoptotic protein Mcl-1 (Figure 4G,H). These findings indicate that the mitochondrial pathway is involved in apoptosis induced by α-MMC.

### 2.5. MAPK Cascade Signaling Was Involved in the Apoptosis-Inducing Effect of α-MMC on MM.1S Cells

**Figure 5:**
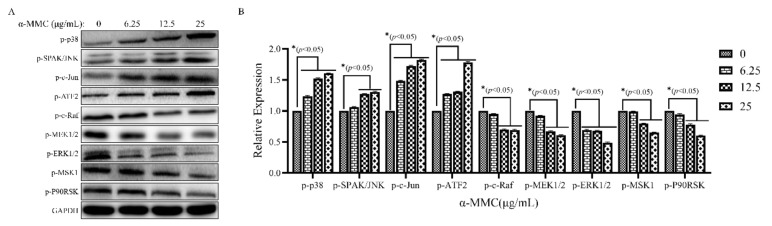
Effect of α-MMC on the MAPK cascade signaling in MM.1S cells. The expression levels of MAPK-signaling-pathway-related proteins in MM.1S cells were detected using Western blot (**A**); GraphPad Prism 9.0 software was used to draw a histogram to reflect the relative expression levels of proteins (**B**). In the experimental group (6.25, 12.5, 25 μg/mL α-MMC group) comparisons with the control group (0 μg/mL α-MMC group), * *p* < 0.05 was considered to be statistically significant.

The mitogen-activated protein kinase (MAPK) signaling pathway involves four protein subfamilies, ERK, p38, JNK, and BMK1, which can be activated by various stimuli, regulate physiological processes, such as cell growth and death, and play important roles in the process of apoptosis. In order to investigate the effect of α-MMC on the MAPK signaling pathway in MM.1S cells, the expression levels of MAPK-signaling-pathway-related proteins were detected via Western blot. The results found that the expressions levels of p-c-Raf, p-MEK1/2, p-ERK1/2, p-MSK1, and p-P90RSK in the MAPK/ERK signaling pathway were significantly decreased, and the expressions levels of p-p38 in the MAPK/p38 signaling pathway and p-SPAK/JNK, p-c-Jun, and p-ATF2 in the MAPK/JNK signaling pathway were significantly increased (Figure 5A,B). The above results suggest that α-MMC may regulate the apoptosis of MM.1S cells by activating MAPK cascade signaling.

## 3. Discussion

RIPs are mainly divided into two types. Type I RIPs are composed of an A chain with RNA N-glycosidase activity, while type II RIPs are heterodimeric proteins which contain an A chain, with enzyme activity, and a B chain, with cell-binding properties, connected by disulfide bonds [9]. Type II RIPs have a higher cytotoxicity than type I, because RIPs must enter cells to exert their RNA N-glycosidase activity to inactivate ribosomes and inhibit protein synthesis. Type II RIPs bind to glycoproteins or glycolipids on the cell membrane through the lectin activity of the B chain, assisting the A chain to enter cells, while type I RIPs have difficulty entering cells without glycol-binding activity, which makes type II extensively cytotoxic [20]. The lack of the B chain significantly restricts the entry of type I RIPs into cells, thereby establishing the high intracellular toxicity and low extracellular toxicity of type I RIPs. This characteristic makes type I RIPs an ideal candidate for targeted tumor therapy, through recombinant fusion or chemical conjugation to tumor-specific ligands or antibodies, which can mediate tumor cell uptake and improve the selective cytotoxicity of type I RIPs [21].

Some studies have found that α-MMC, as a type I RIP, can enter cells to exert glycosidase activity and inactivate ribosomes. The main mechanism by which this occurs is that α-MMC specifically binds to low-density lipoprotein receptor-related protein 1 (LRP1), which produces cytotoxicity through LRP1-mediated endocytosis and JNK signal transduction activity [18,22]. LRP1 is a transmembrane receptor that can mediate the endocytosis of different ligands. LRP1 has signal transduction activity and is widely distributed on the surface of tumor cells, such as myeloma, breast cancer, glioma, and melanoma cells [18,23]. In this study, the CCK-8 assay showed that α-MMC inhibited the proliferation of myeloma MM.1S cells in a time-dependent and drug-concentration-dependent manner, with IC_50_ values of 13.04 and 7.518 μg/mL for 24 and 48 h, respectively. At the same time, the results of the CCK-8 showed that α-MMC had no significant cytotoxicity to normal human PBMCs within the effective concentration range. Further exploring the mechanism of the antiproliferation effect of α-MMC on MM.1S cells, it was found that α-MMC could arrest the MM.1S cell cycle at the G2 phase and increase the proportion of sub-G0/G1 cells. Based on this, we speculated that α-MMC can arrest the MM.1S cell cycle and induce apoptosis.

Apoptosis is a form of programmed cell death, which can eliminate harmful or damaged cells in the body in an orderly and effective manner [24]. Apoptosis can be triggered via both endogenous and exogenous pathways, such as oxidative stress and the binding of ligands to cell surface death receptors. These two pathways converge to activate effector molecules related to apoptosis, namely caspase protease, which ultimately leads to changes in cell morphology and biochemistry, which are the characteristics of cell apoptosis [25]. The caspase family is the core of the occurrence and development of apoptosis. Caspase-2, -8, -9, and -10 are the initiators of apoptosis, and caspase-3, -6, and -7 are the executors of apoptosis [25,26]. They can cleave specific substrates, such as poly ADP ribose polymerase (PARP). PARP is a DNA-repair enzyme, which plays an important role in the process of DNA damage repair and cell apoptosis. PARP can be cut into 89 kDa fragments by a variety of caspases, which is conducive to the spread of apoptotic death stimuli [27]. The Bcl-2 protein family can regulate the integrity and function of the mitochondrial outer membrane and participate in the reception and transmission of apoptosis signals [28,29]. After receiving the apoptosis signal, the pro-apoptotic protein Bax undergoes a conformational change and translocates from the cytoplasm to the mitochondrial outer membrane, which expands the permeability pores on the membrane, causing a decrease in MMP and the release of pro-apoptotic substances such as AIF and Cyt-c, and then initiates the caspase cascade reaction, cleaves PARP and other substrates, and, finally, induces apoptosis [30]. The pro-apoptotic protein Bid contains only the BH3 domain and promotes apoptosis by directly binding to Bax [31]. Mcl-1 exerts its anti-apoptotic effect, on the one hand, by binding to BH3-only proteins and preventing them from activating Bax or Bak proteins, and, on the other hand, by directly binding to Bak proteins on the mitochondrial membrane [32]. The results of the FCM detection and analysis showed that after 25 μg/mL α-MMC treatment for 24 h, the apoptosis rate of the MM.1S cells increased from 6.99 ± 1.26% to 62.33 ± 3.85%, and the change rate of MMP increased from 6.44 ± 0.08% to 62.23 ± 4.24%. In addition, the Western blot results showed that α-MMC upregulated the expressions levels of Bax, Bid, cleaved caspase-3, and cleaved PARP and downregulated the expression levels of the anti-apoptotic protein Mcl-1. The above results suggest that α-MMC induces apoptosis of MM.1S cells through the mitochondrial pathway.

MM is a neoplastic disease with high levels of heterogeneity, which is mostly driven by genetic factors [33]. In 1273 newly diagnosed patients with MM, 63 different driver genes were identified using genomic analysis [34]. These driver genes accelerate tumorigenesis by inducing abnormal expression of downstream signaling pathways, including the MAPK signaling pathway. MAPK is the hub of multiple signaling pathways. The MAPK/ERK pathway is involved in cell proliferation and differentiation, and its overactivation plays an important role in the occurrence and development of tumors [35]. The MAPK/p38 and MAPK/JNK families are closely related to cell death, and their silencing can inhibit cell apoptosis [36]. Therapies that target these abnormally expressed signaling pathways have yielded impressive results in malignancies [37,38,39]. After the treatment of α-MMC, the expression levels of the MAPK-signaling-pathway-related proteins were detected using Western blot. The results showed that the expression levels of p-c-Raf, p-MEK1/2, p-ERK1/2, p-MSK1, and p-P90RSK in the MAPK/ERK signaling pathway significantly decreased, while the expression levels of p-p38 in the MAPK/p38 signaling pathway and the expressions levels of p-SPAK/JNK, p-c-Jun, and p-ATF2 in the MAPK/JNK signaling pathway significantly increased. These results suggest that α-MMC induced the apoptosis of MM.1S cells by activating MAPK cascade signaling.

## 4. Materials and Methods

### 4.1. Materials

The α-MMC was donated by Yanfa Meng from the College of Life Sciences of Sichuan University [19]. The RPMI-1640 basal medium and fetal bovine serum (FBS) were from Gibco (Grand Island, NE, USA). The Lymphoprep™ separation solution was purchased from Axis-Shield PoC (Oslo, Norway). The CCK-8 was obtained from Dojindo (Kumamoto Prefecture, Japan). The mitochondrial membrane potential assay kit with JC-1, annexin V-FITC/PI apoptosis detection kit, cell cycle and apoptosis analysis kit, and RIPA lysis buffer were from Beyotime Biotechnology (Shanghai, China). The PhosSTOP phosphatase inhibitor cocktail and complete™ protease inhibitor cocktail were obtained from Roche (Basel, Switzerland). The BSA albumin fraction V and ultra-sensitive ECL chemiluminescence substrate were from Biosharp (Hefei, China). The BCA protein quantitative kit was obtained from Vazyme (Nanjing, China). The antibodies against cleaved caspase-3, cleaved PARP, Bax, Bid, Mcl-1, p-p38, p-SPAK/JNK, p-c-Jun, p-ATF2, p-c-Raf, p-MEK1/2, p-ERK1/2, p-MSK1, p-P90RSK, GAPDH, and HRP-conjugated goat antirabbit antibody were purchased from CST (Boston, MA, USA). All other chemicals were of analytical grade.

### 4.2. Cell Culture

Multiple myeloma MM.1S cells were purchased from the American Type Culture Collection (ATCC) (Manassas, VA, USA) and cryopreserved in a liquid nitrogen tank in the laboratory. According to the culture method recommended by ATCC, the MM.1S cells were cultured in RPMI-1640 medium containing 10% FBS at 37 °C with 5% CO_2_. When MM.1S cells were growing well and in logarithmic growth phase, they were used for subsequent experiments.

### 4.3. Separation of PBMCs

Peripheral whole blood samples were collected from healthy people using vacuum blood collection tubes containing ethylene diamine tetra acetic acid anticoagulant and diluted with equal amount of phosphate buffer solution (PBS). A centrifuge tube containing Lymphoprep™ separation solution was tilted 45°, and the diluted peripheral whole blood was slowly added along the tube wall to ensure that the diluted whole blood did not enter the Lymphoprep™ separation solution. After centrifugation, the PBMCs in the middle layer were collected and washed twice via centrifugation with PBS, and then the PBMCs were cultured in RPMI-1640 medium containing 10% FBS. 

### 4.4. Cell Proliferation and Cytotoxicity Assay

MM.1S cells and PBMCs were inoculated separately and at the same density into 96-well cell culture plates. Subsequently, α-MMC at a concentration of 0, 1.5625, 3.125, 6.25, 12.5, 25, and 50 μg/mL was added and plates were incubated for 24 or 48 h. CCK-8 solution was added to each well and incubated for another 4 h. The absorbance at 450 nm was measured using a multimode plate reader (PerkinElmer VICTOR Nivo, Waltham, MA, USA). GraphPad Prism 9.0 software was used to draw the growth curves of the MM.1S cells and PBMCs after their treatment with α-MMC and to calculate the IC_50_ value.

### 4.5. Cell Cycle Assay

After being treated with 0, 3.125, 6.25, and 12.5 μg/mL α-MMC for 12 h, the MM.1S cells were collected by centrifugation. The cell precipitates were resuspended with 300 μL precooled PBS, added drip by drip to 700 μL precooled anhydrous ethanol on a vortex mixer (Kylin-Bell Lab Instruments XW-80A, Haimen, China), and then fixed at 4 °C overnight. After the ethanol was removed by centrifugation, the cells were resuspended with staining solution containing staining buffer, PI, and RNase-A according to the kit instructions. After staining in the dark for 30 min, the cells were collected using FCM (ACEA Biosciences NovoCyte Quanteon, San Diego, CA, USA) for cell cycle distribution analysis.

### 4.6. Cell Apoptosis Assay

After treatment with α-MMC for 24 h, the morphological changes of the MM.1S cells were observed and imaged using an inverted microscope (Olympus IX83, Tokyo, Japan). Subsequently, the MM.1S cells were collected by centrifugation and resuspended in stained buffer containing annexin V-FITC and PI. After staining in the dark for 20 min, FCM was used to detect and analyze the apoptosis-inducing effect of α-MMC on the MM.1S cells.

### 4.7. Mitochondrial Membrane Potential Assay

To explore the effect of α-MMC on the MMP of MM.1S cells, the MM.1S cells treated with α-MMC for 24 h were collected by centrifugation, and resuspended with staining buffer containing JC-1 fluorescent probe. After staining in the dark for 20 min at 37 °C, FCM was used to detect and analyze the proportion of MM.1S cells in which the red fluorescence had shifted to green fluorescence to reflect the change in the MMP in the MM.1S cells.

### 4.8. Western Blot Analysis

After being treated with α-MMC for 24 h, MM.1S cells were lysed in RIPA lysis buffer containing phosphatase inhibitor and protease inhibitor, and the lysates were centrifuged at 13,000 rpm for 20 min at 4 °C. The protein concentration was determined using the BCA method. The protein mass, volume, and concentration were adjusted to the same levels, and 50 μg measures of total proteins were separated into proteins of different molecular weights by 12% sodium dodecyl sulfate polyacrylamide gel electrophoresis (SDS-PAGE). Then, the proteins were transferred to polyvinylidene fluoride membranes (PVDF) via wet electro-transfer at 250 mA. The membranes were blocked for 1 h with 5% BSA at 37 °C and incubated overnight at 4 °C with the respective primary antibodies, followed by incubation with the secondary antibody conjugated to horseradish peroxidase (HRP) for 2 h. The blots were visualized via the ultrasensitive ECL chemiluminescence substrate, enhanced chemiluminescence system (Bio-Rad ChemiDocXRS, Hercules, CA, USA), and Quantity-One image acquisition software. The integrated optical density (IOD) values of the protein blot were analyzed via Image-J analysis software. GAPDH was used as the internal reference protein, and the target protein’s IOD/internal reference protein’s IOD ratio reflecting the relative expression levels of the target protein.

### 4.9. Statistical Analysis 

Statistical analysis was performed using the GraphPad Prism 9.0 software. Each group of experiments was independently repeated three times, and the experimental results were expressed as the mean ± standard deviation. The differences between multiple groups were compared using one-way ANOVA and the Dunnett *t*-test. In comparisons with the control group, *p* < 0.05 was considered to be statistically significant.

## 5. Conclusions

It has been found that there are five types of MMC, among which α-MMC and β-MMC have been more intensively studied because of their effective biological activities. Meanwhile, in terms of the extraction and purification process from the edible plant *Momordica charantia* L., α-MMC tends to have higher yields and more stable enzymatic activity compared to β-MMC [40]. Our findings suggest that α-MMC has an inhibitory effect on the proliferation of multiple myeloma MM.1S cells in vitro. α-MMC induces apoptosis by arresting the cell cycle, reducing MMP, triggering mitochondrial apoptosis pathways, and activating MAPK cascade signaling. The research results elucidate the molecular mechanism of α-MMC as a new candidate drug for multiple myeloma and expand the application possibilities of ribosome-inactivating proteins from edible plants.

## Data Availability

Data is contained within article and Supplementary Materials.

## Supplementary Materials

The following supporting information can be downloaded at: https://www.mdpi.com/article/10.3390/ph16010124/s1.
