# Supplementary material for: MAPK Cascade Signaling Is Involved in α-MMC Induced Growth Inhibition of Multiple Myeloma MM.1S Cells via G2 Arrest and Mitochondrial-Pathway-Dependent Apoptosis In Vitro
Source: Pharmaceuticals (Basel). 2023 Jan 13;16(1):124. doi: 10.3390/ph16010124 (PMC9867419; doi:10.3390/ph16010124)
Supplement: Supplementary file 1 [file pharmaceuticals-16-00124-s001.zip › FCM analysis imaging DW.pdf]

Report of α-MMC(μg/mL)+MM.1S DW

Specimen Name: α-MMC(μg/mL)+MM.1S DW

Run Time: 2022/3/14 19:37

Cytometer: NovoCyte Quanteon 622181010270

Software: NovoExpress 1.4.0

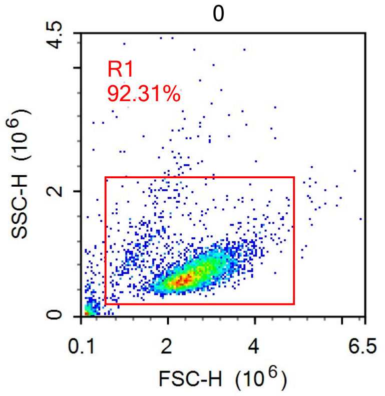

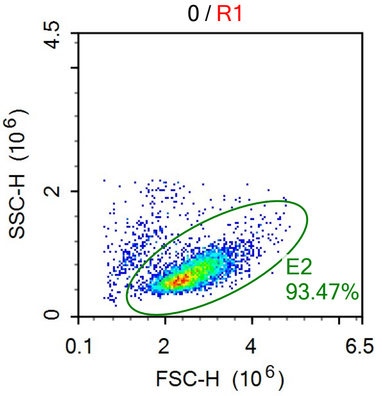

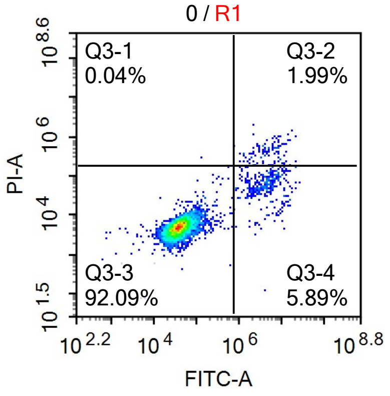

Sample Statistics of 0

Gate	Count	% Parent	X	Y	Median X	Median Y
All	5,722					
R1	5,282	92.31%	FSC-H	SSC-H	2,509,583	654,008
E2	4,937	93.47%	FSC-H	SSC-H	2,543,310	640,792
Q3-1	2	0.04%	FITC-A	PI-A	424,916	443,592
Q3-2	105	1.99%	FITC-A	PI-A	4,648,527	439,044
Q3-3	4,864	92.09%	FITC-A	PI-A	36,204	4,975
Q3-4	311	5.89%	FITC-A	PI-A	4,354,817	58,561

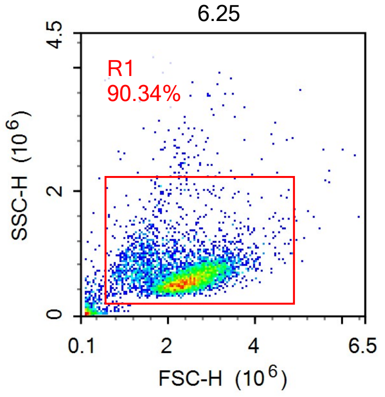

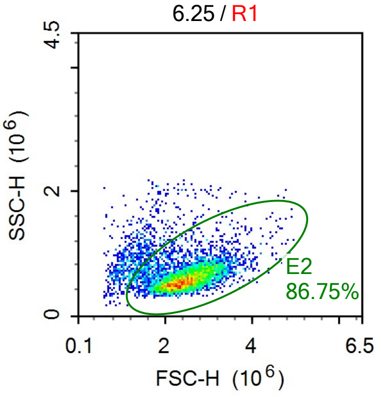

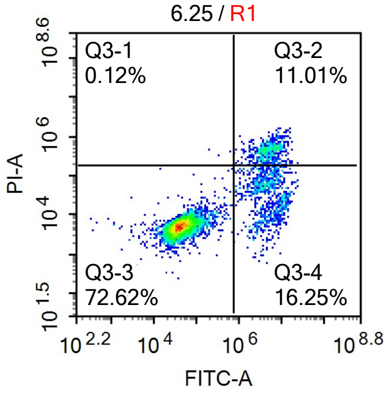

Sample Statistics of 6.25

Gate	Count	% Parent	X	Y	Median X	Median Y
All	6,213					
R1	5,613	90.34%	FSC-H	SSC-H	2,395,605	607,461
E2	4,869	86.75%	FSC-H	SSC-H	2,481,630	582,859
Q3-1	7	0.12%	FITC-A	PI-A	687,487	373,024
Q3-2	618	11.01%	FITC-A	PI-A	4,797,816	460,641
Q3-3	4,076	72.62%	FITC-A	PI-A	39,409	5,175
Q3-4	912	16.25%	FITC-A	PI-A	4,575,498	36,776

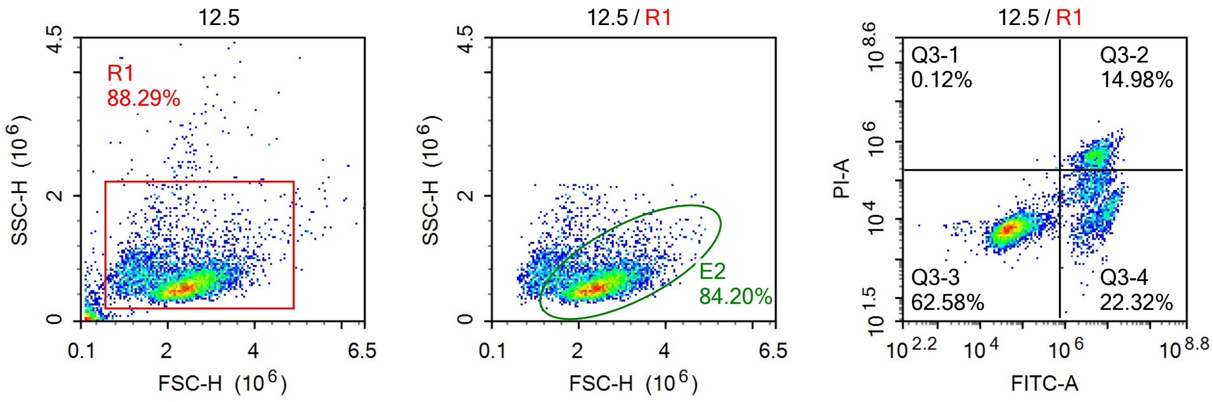

Sample Statistics of 12.5

Gate	Count	% Parent	X	Y	Median X	Median Y
All	6,501					
R1	5,740	88.29%	FSC-H	SSC-H	2,355,941	597,902
E2	4,833	84.20%	FSC-H	SSC-H	2,451,786	567,484
Q3-1	7	0.12%	FITC-A	PI-A	566,383	253,630
Q3-2	860	14.98%	FITC-A	PI-A	5,196,987	440,036
Q3-3	3,592	62.58%	FITC-A	PI-A	46,367	5,994
Q3-4	1,281	22.32%	FITC-A	PI-A	5,150,986	28,843

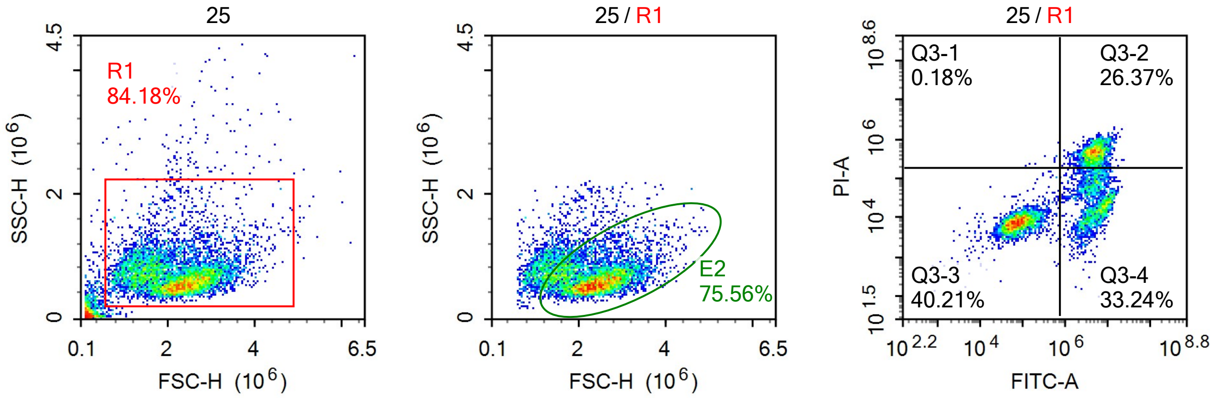

Sample Statistics of 25

Gate	Count	% Parent	X	Y	Median X	Median Y
All	7,451					
R1	6,272	84.18%	FSC-H	SSC-H	2,208,089	665,919
E2	4,739	75.56%	FSC-H	SSC-H	2,413,784	616,906
Q3-1	11	0.18%	FITC-A	PI-A	521,723	308,649
Q3-2	1,654	26.37%	FITC-A	PI-A	4,807,298	458,835
Q3-3	2,522	40.21%	FITC-A	PI-A	70,945	7,577
Q3-4	2,085	33.24%	FITC-A	PI-A	4,734,268	24,892
