# Supplementary material for: MAPK Cascade Signaling Is Involved in α-MMC Induced Growth Inhibition of Multiple Myeloma MM.1S Cells via G2 Arrest and Mitochondrial-Pathway-Dependent Apoptosis In Vitro
Source: Pharmaceuticals (Basel). 2023 Jan 13;16(1):124. doi: 10.3390/ph16010124 (PMC9867419; doi:10.3390/ph16010124)
Supplement: Supplementary file 1 [file pharmaceuticals-16-00124-s001.zip › FCM analysis imaging MMP.pdf]

# Report of α-MMC(μg/mL)+MM.1S MMP

Specimen Name: α-MMC(μg/mL)+MM.1S MMP | Run Time: 2022/8/4 15:30

Cytometer: NovoCyte Quanteon 622181010270 | Software: NovoExpress 1.4.0

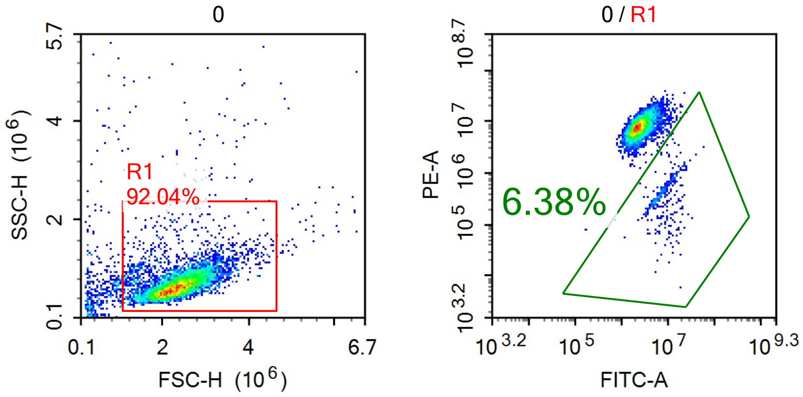


Sample Statistics of 0

| Gate | Count | % Parent | X | Y | Median X | Median Y |
|---|---|---|---|---|---|---|
| All | 6,408 | | | | | |
| R1 | 5,898 | 92.04% | FSC-H | SSC-H | 2,315,401 | 670,170 |
| P2 | 376 | 6.38% | FITC-A | PE-A | 7,686,580 | 339,277 |

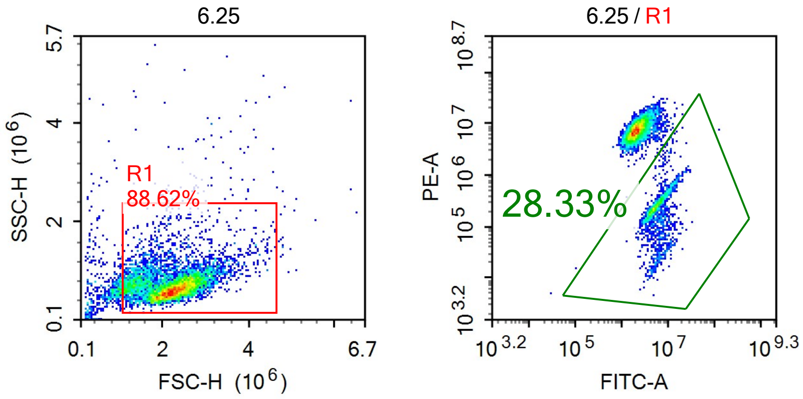


Sample Statistics of 6.25

| Gate | Count | % Parent | X | Y | Median X | Median Y |
|---|---|---|---|---|---|---|
| All | 6,589 | | | | | |
| R1 | 5,839 | 88.62% | FSC-H | SSC-H | 2,204,367 | 704,390 |
| P2 | 1,654 | 28.33% | FITC-A | PE-A | 5,723,024 | 208,501 |

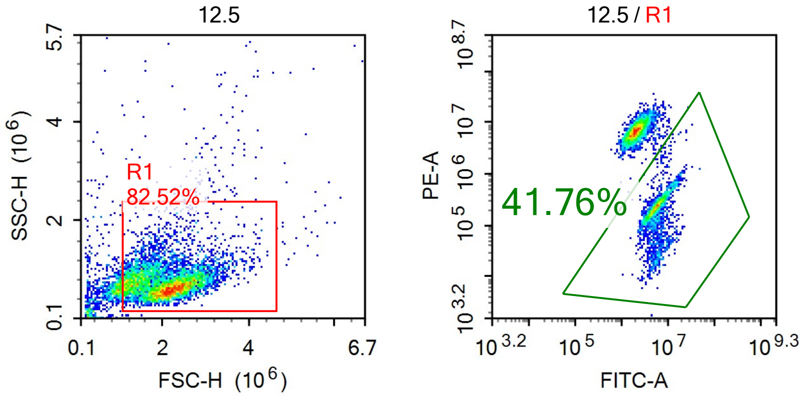


Sample Statistics of 12.5

| Gate | Count | % Parent | X | Y | Median X | Median Y |
|---|---|---|---|---|---|---|
| All | 6,901 | | | | | |
| R1 | 5,695 | 82.52% | FSC-H | SSC-H | 2,063,356 | 732,805 |
| P2 | 2,378 | 41.76% | FITC-A | PE-A | 5,462,342 | 199,063 |

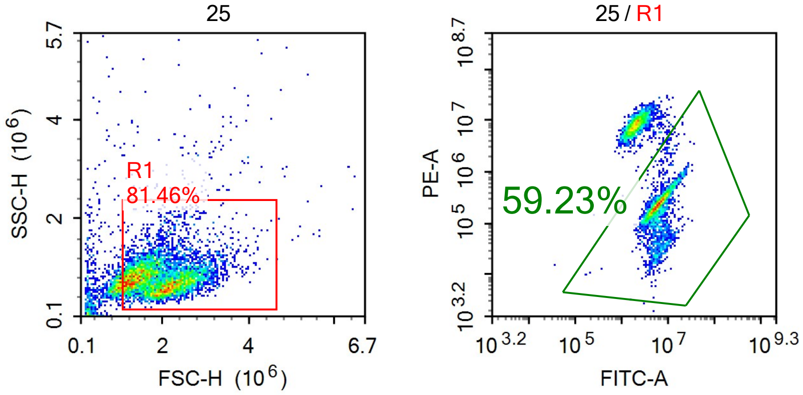


Sample Statistics of 25

| Gate | Count | % Parent | X | Y | Median X | Median Y |
|---|---|---|---|---|---|---|
| All | 6,926 | | | | | |
| R1 | 5,642 | 81.46% | FSC-H | SSC-H | 1,889,254 | 793,813 |
| P2 | 3,342 | 59.23% | FITC-A | PE-A | 5,880,032 | 205,434 |
