# Supplementary material for: MAPK Cascade Signaling Is Involved in α-MMC Induced Growth Inhibition of Multiple Myeloma MM.1S Cells via G2 Arrest and Mitochondrial-Pathway-Dependent Apoptosis In Vitro
Source: Pharmaceuticals (Basel). 2023 Jan 13;16(1):124. doi: 10.3390/ph16010124 (PMC9867419; doi:10.3390/ph16010124)
Supplement: Supplementary file 1 [file pharmaceuticals-16-00124-s001.zip › FCM analysis imaging ZQ.pdf]

Report of α-MMC(μg/mL)+MM.1S ZQ

Specimen Name: α-MMC(μg/mL)+MM.1S ZQ | Run Time: 2022/10/12 10:37

Cytometer: NovoCyte Quanteon 622181010270 | Software: NovoExpress 1.4.0

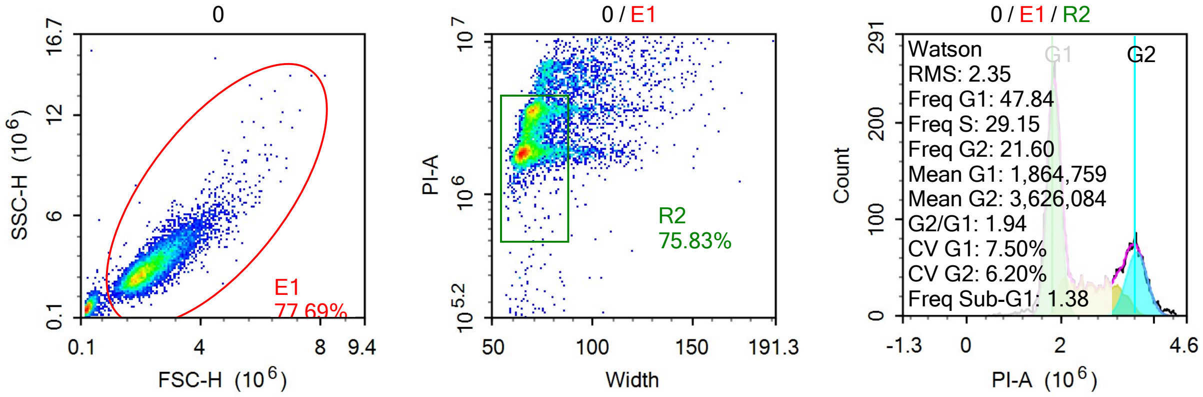

Sample Statistics of 0

Gate	Count	% Parent	X	Y	Median X	Median Y
All	12,871					
E1	10,000	77.69%	FSC-H	SSC-H	2,250,760	2,831,502
R2	7,583	75.83%	Width	PI-A	68	2,092,946

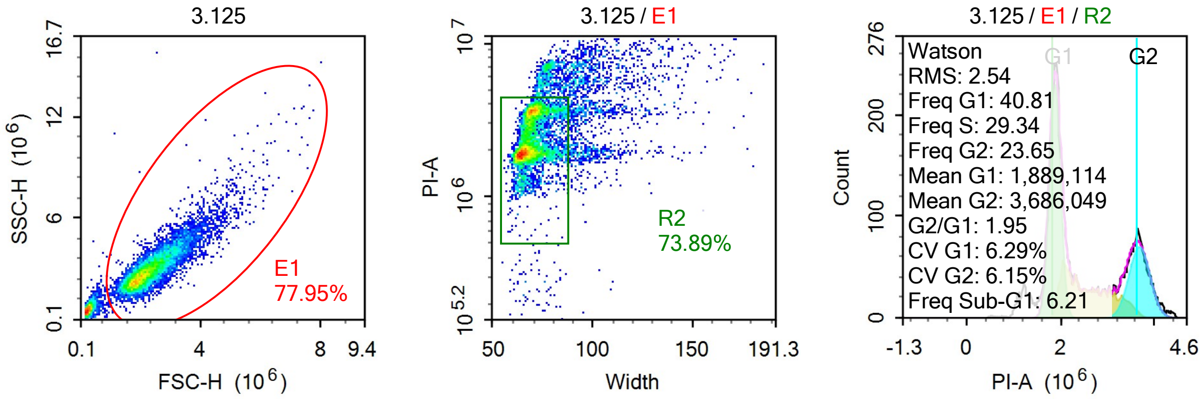

Sample Statistics of 3.125

Gate	Count	% Parent	X	Y	Median X	Median Y
All	12,828					
E1	10,000	77.95%	FSC-H	SSC-H	2,253,617	2,810,742
R2	7,389	73.89%	Width	PI-A	68	2,096,676

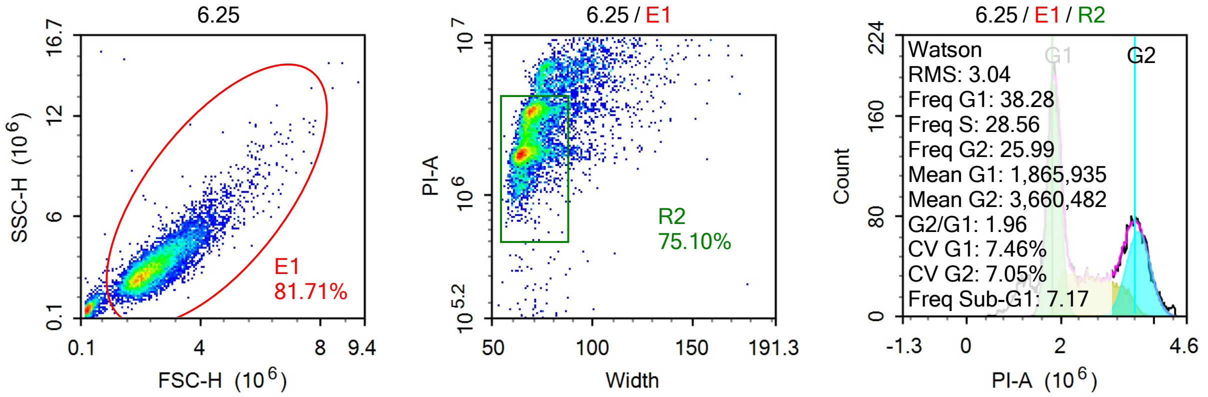

Sample Statistics of 6.25

Gate	Count	% Parent	X	Y	Median X	Median Y
All	12,239					
E1	10,000	81.71%	FSC-H	SSC-H	2,362,509	2,874,632
R2	7,510	75.10%	Width	PI-A	67	2,155,898

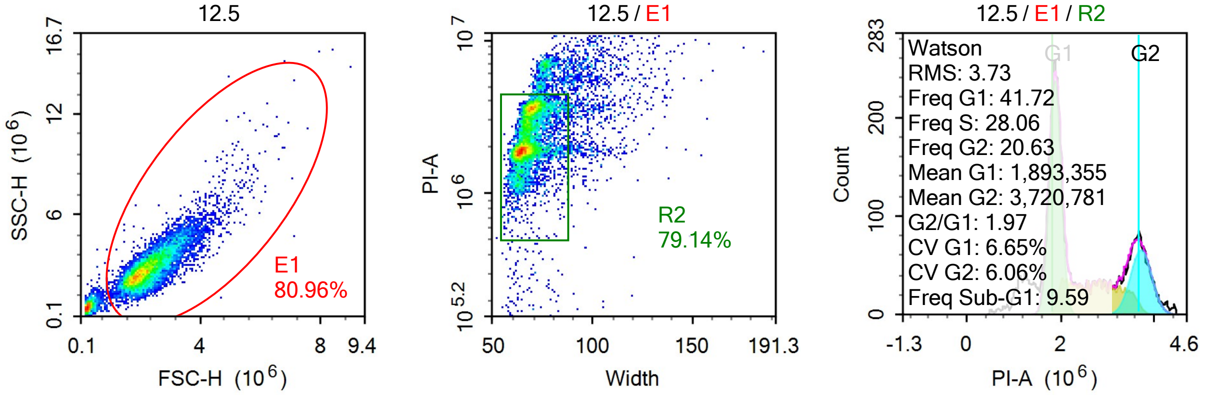

Sample Statistics of 12.5

Gate	Count	% Parent	X	Y	Median X	Median Y
All	12,352					
E1	10,000	80.96%	FSC-H	SSC-H	2,246,043	2,729,576
R2	7,914	79.14%	Width	PI-A	67	2,050,537
