# Supplementary material for: MAPK Cascade Signaling Is Involved in α-MMC Induced Growth Inhibition of Multiple Myeloma MM.1S Cells via G2 Arrest and Mitochondrial-Pathway-Dependent Apoptosis In Vitro
Source: Pharmaceuticals (Basel). 2023 Jan 13;16(1):124. doi: 10.3390/ph16010124 (PMC9867419; doi:10.3390/ph16010124)

Fig4-Bax α-MMC(μg/mL)： 0-6.25-12.5-25

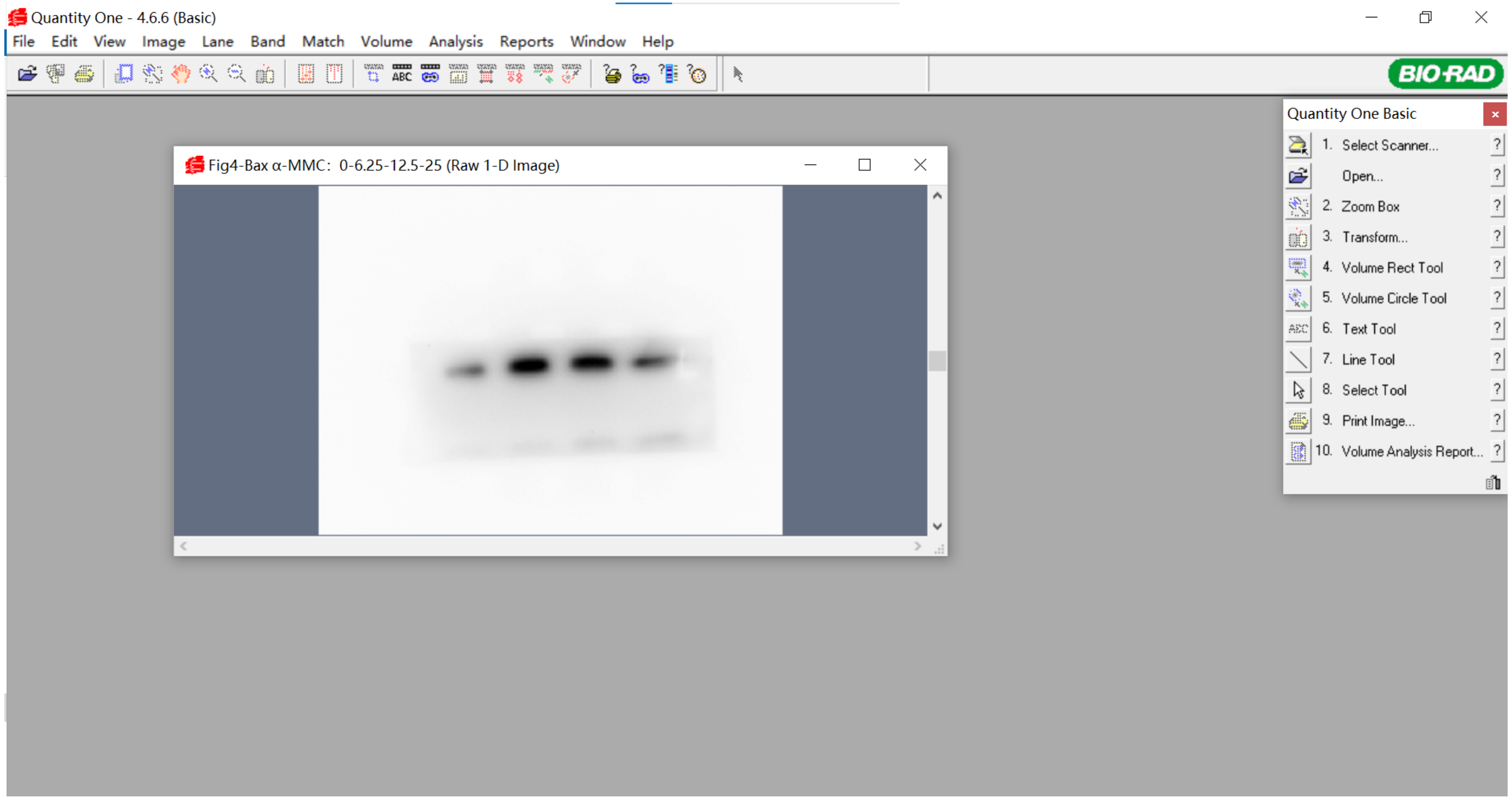

Fig4-Bid α-MMC (μg/mL)： 0-6.25-12.5-25

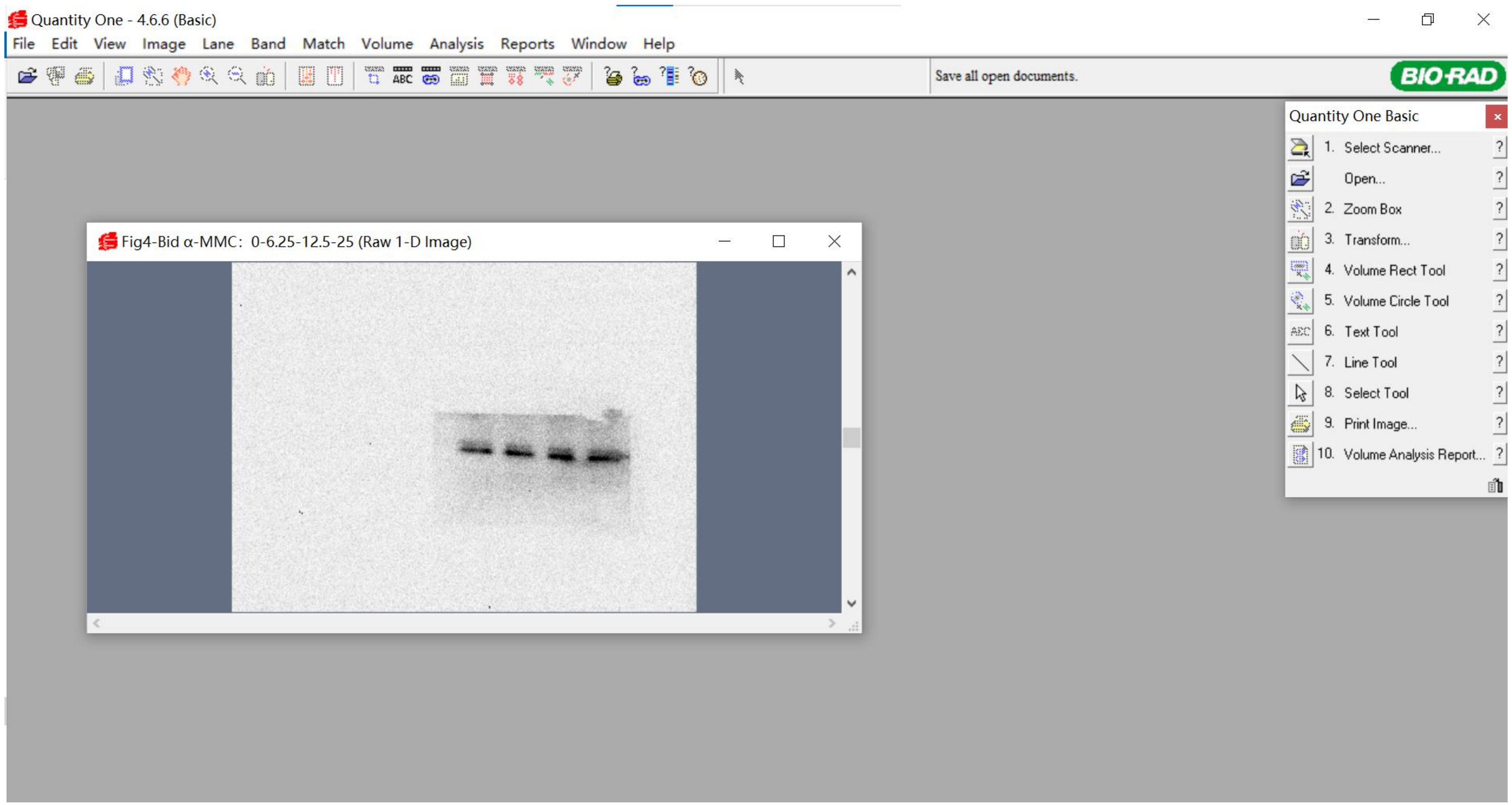

Fig4-cleaved caspase-3 α-MMC (μg/mL)： 0-6.25-12.5-25

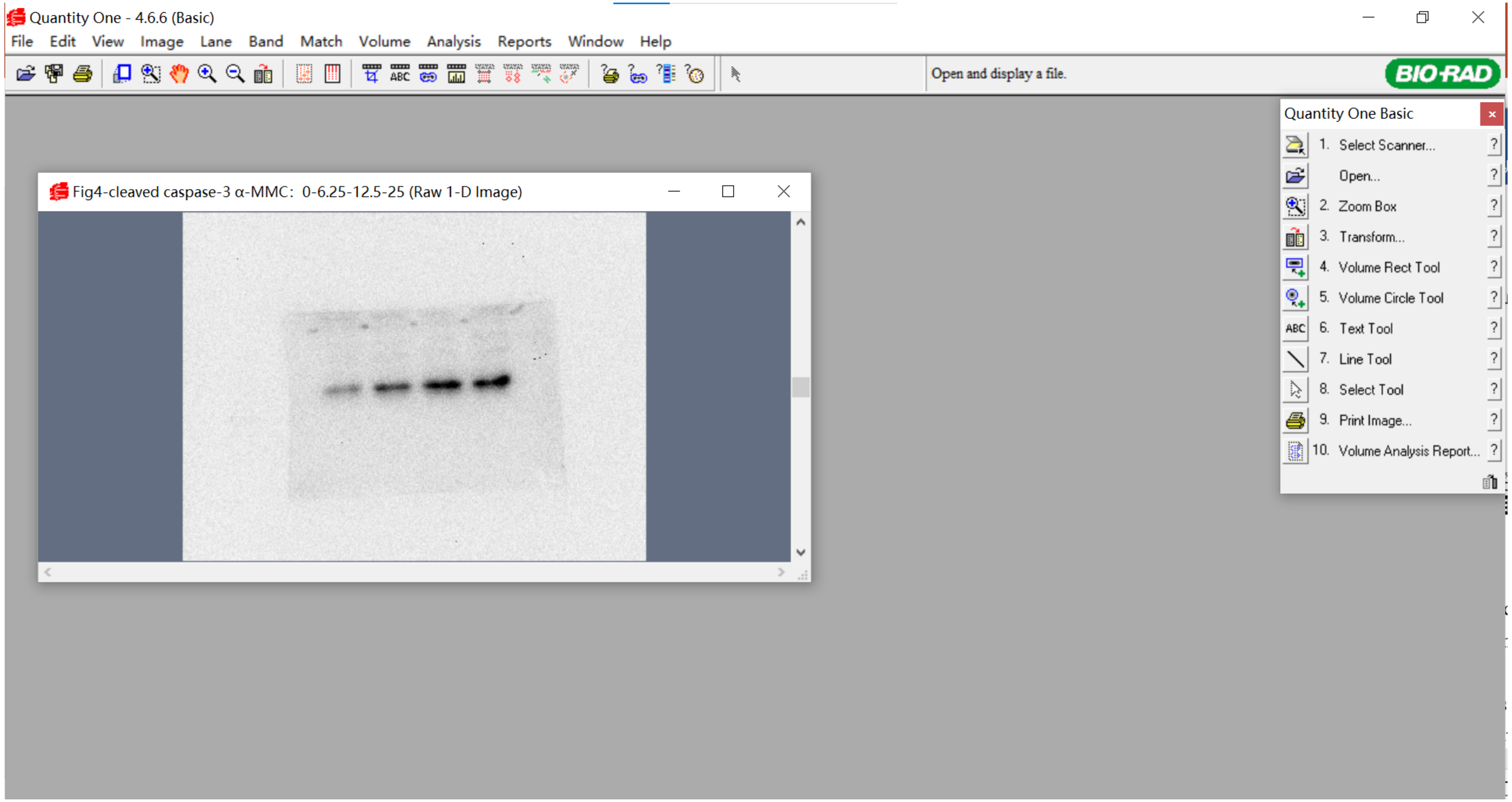

Fig4-cleaved PARP α-MMC (μg/mL)： 0-6.25-12.5-25

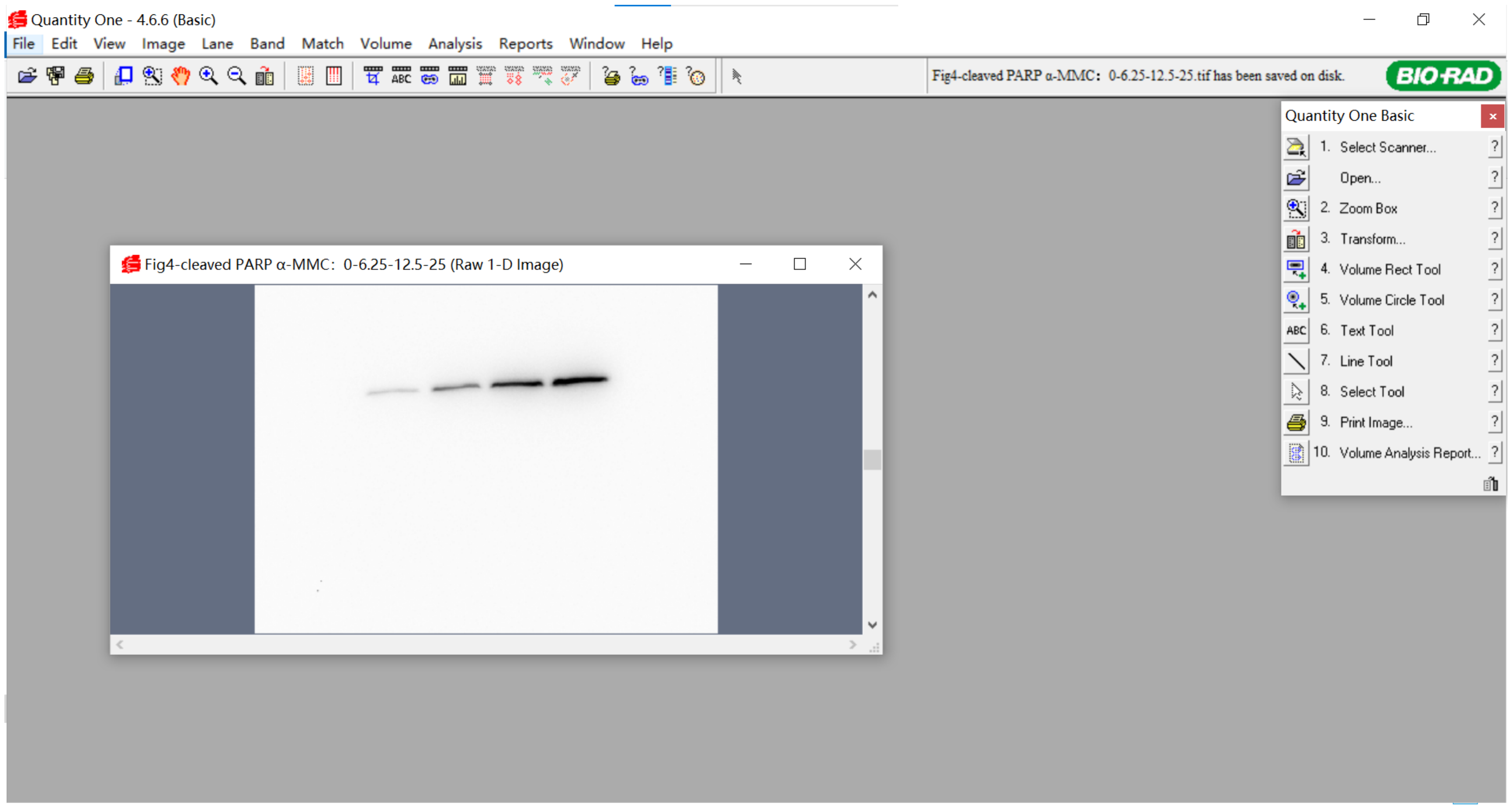

Fig4-Mcl-1 α-MMC (μg/mL)： 0-6.25-12.5-25

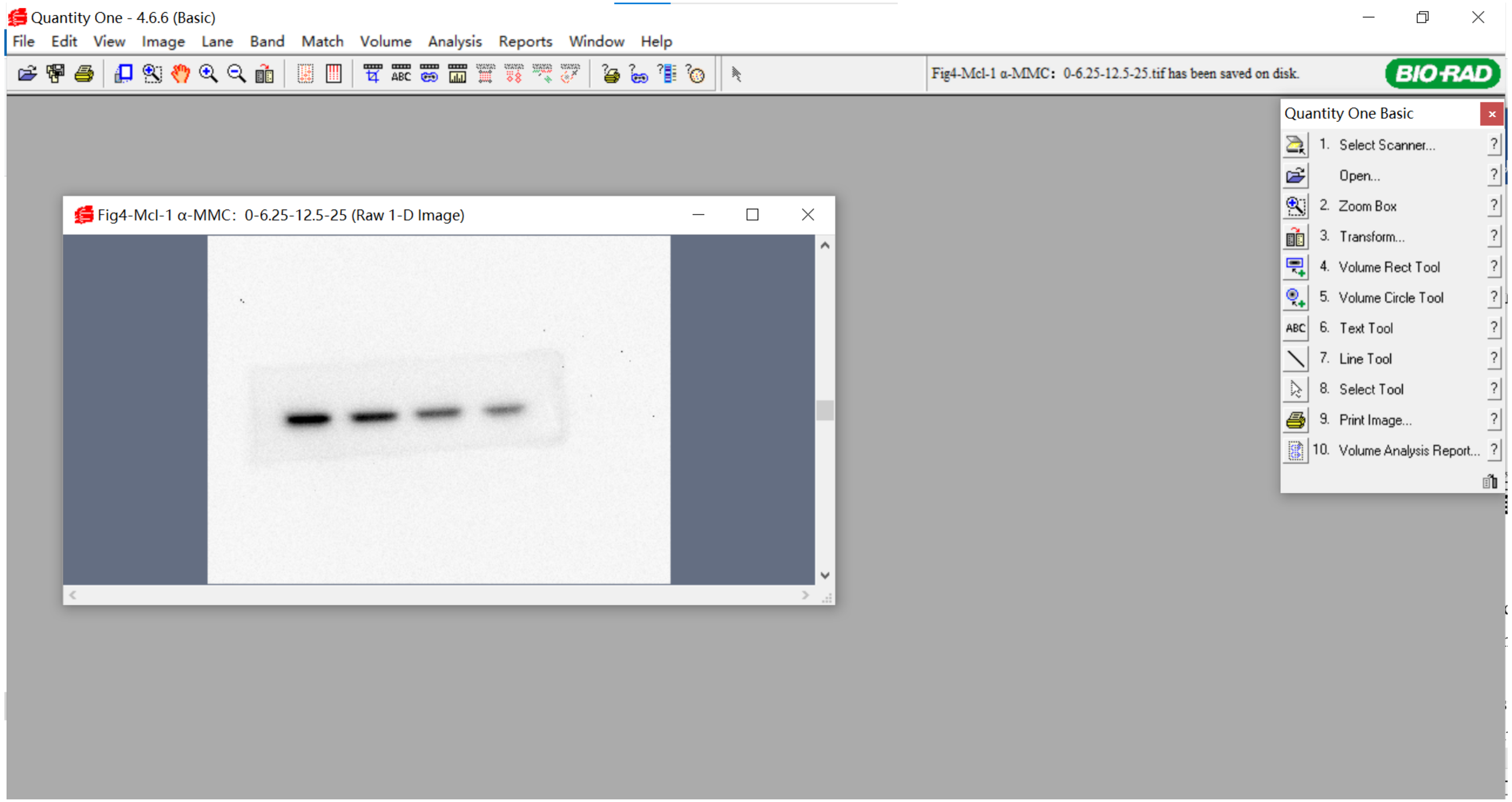

Fig5-p-ATF2 α-MMC (μg/mL)： 0-6.25-12.5-25

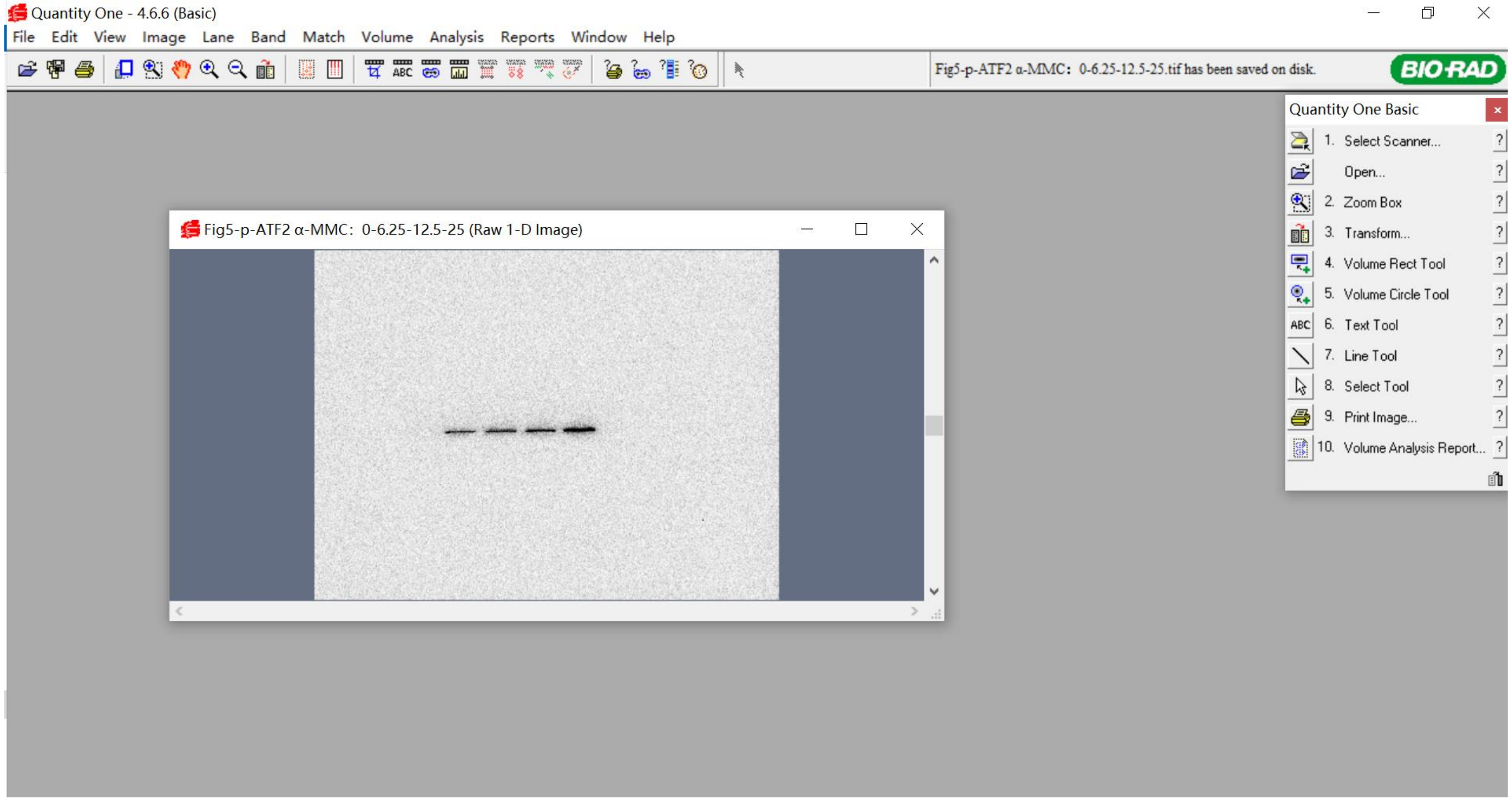

Fig5-p-c-Jun α-MMC (μg/mL)： 0-6.25-12.5-25-50

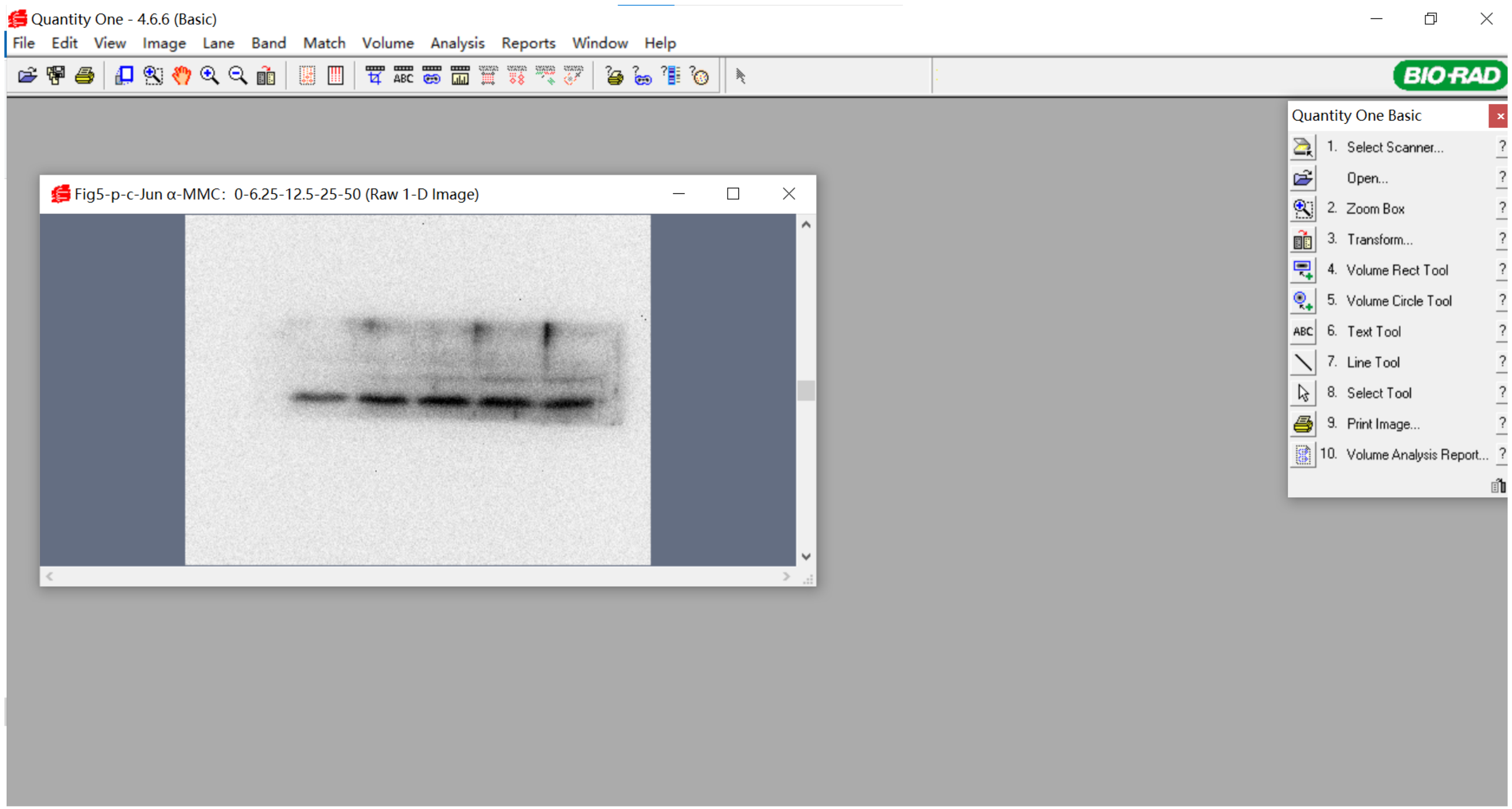

Fig5-p-c-Raf α-MMC (μg/mL)： 0-6.25-12.5-25

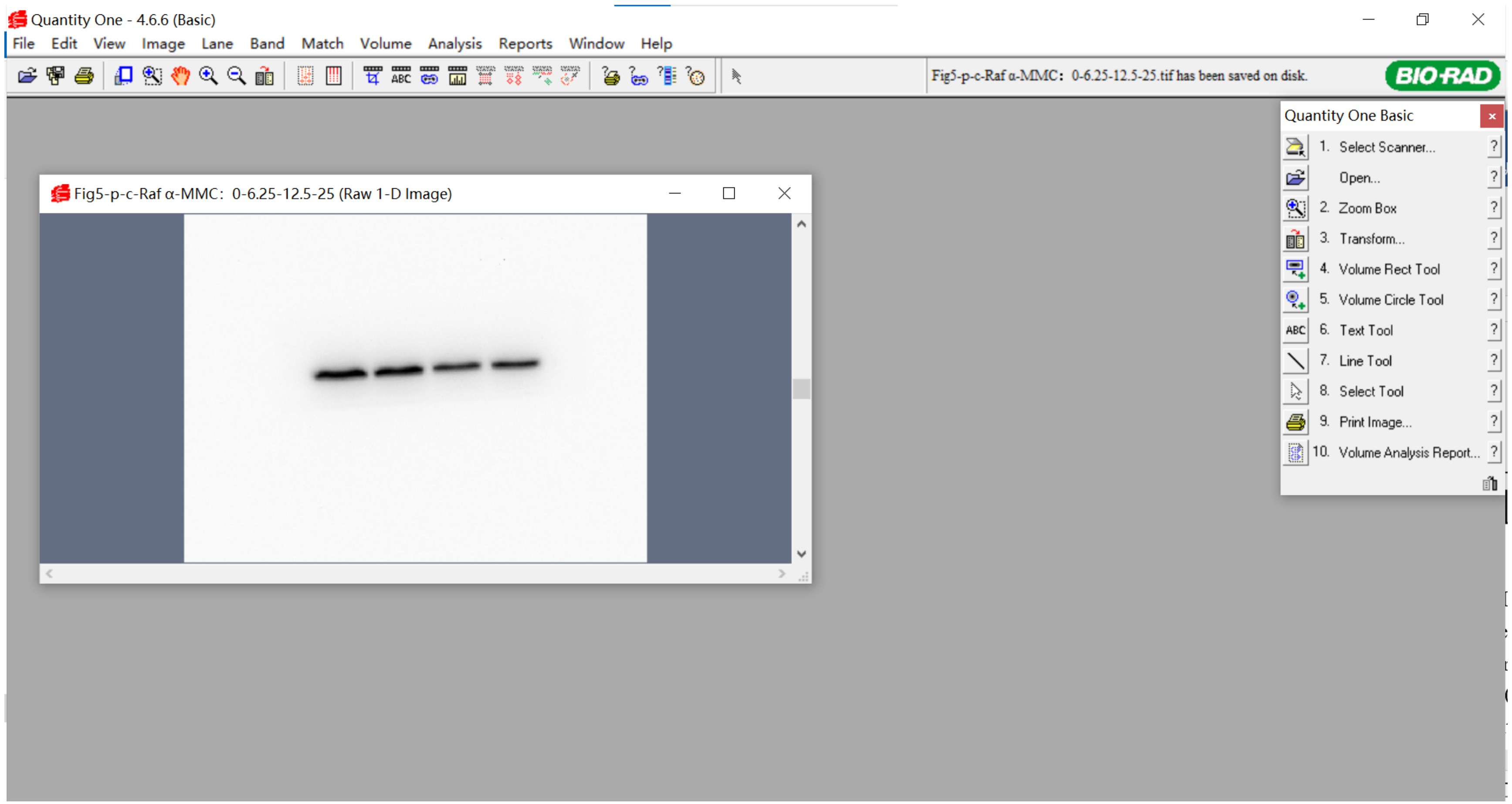

Fig5-p-ERK1/2 α-MMC (μg/mL)： 0-6.25-12.5-25

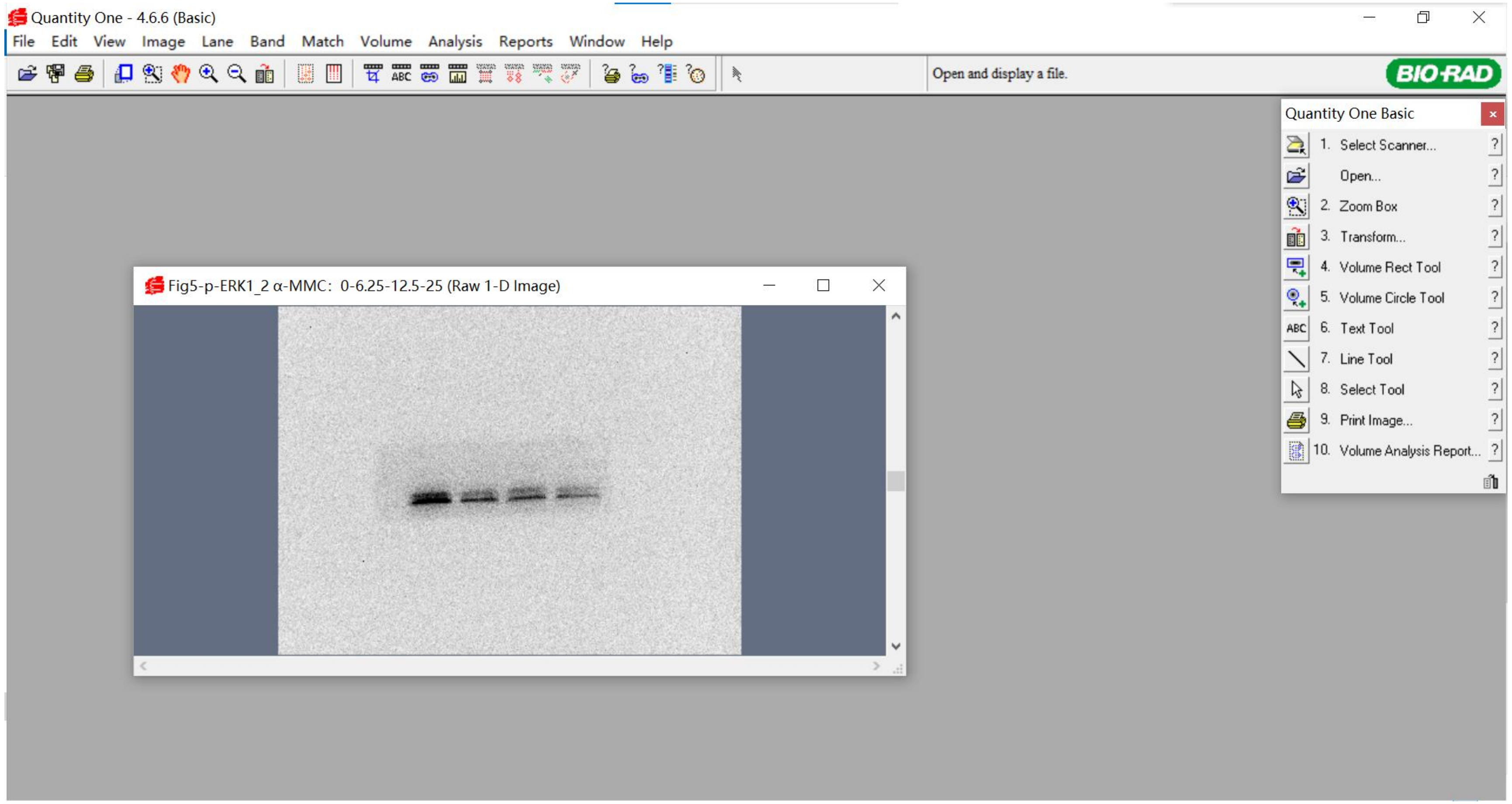

Fig5-p-MEK1_2 α-MMC (μg/mL)： 0-6.25-12.5-25

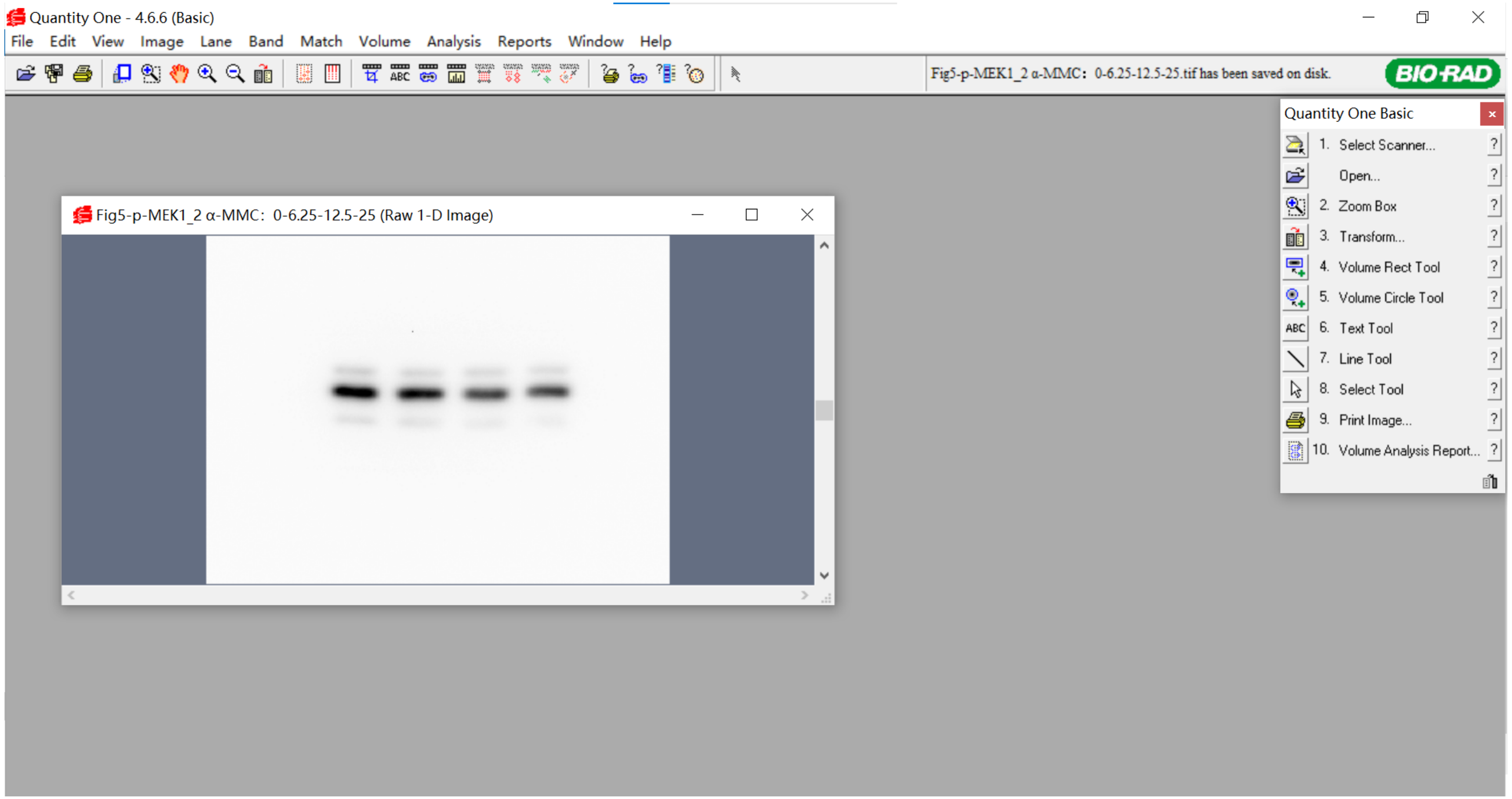

Fig5-p-MSK1 α-MMC (μg/mL)： 0-6.25-12.5-25

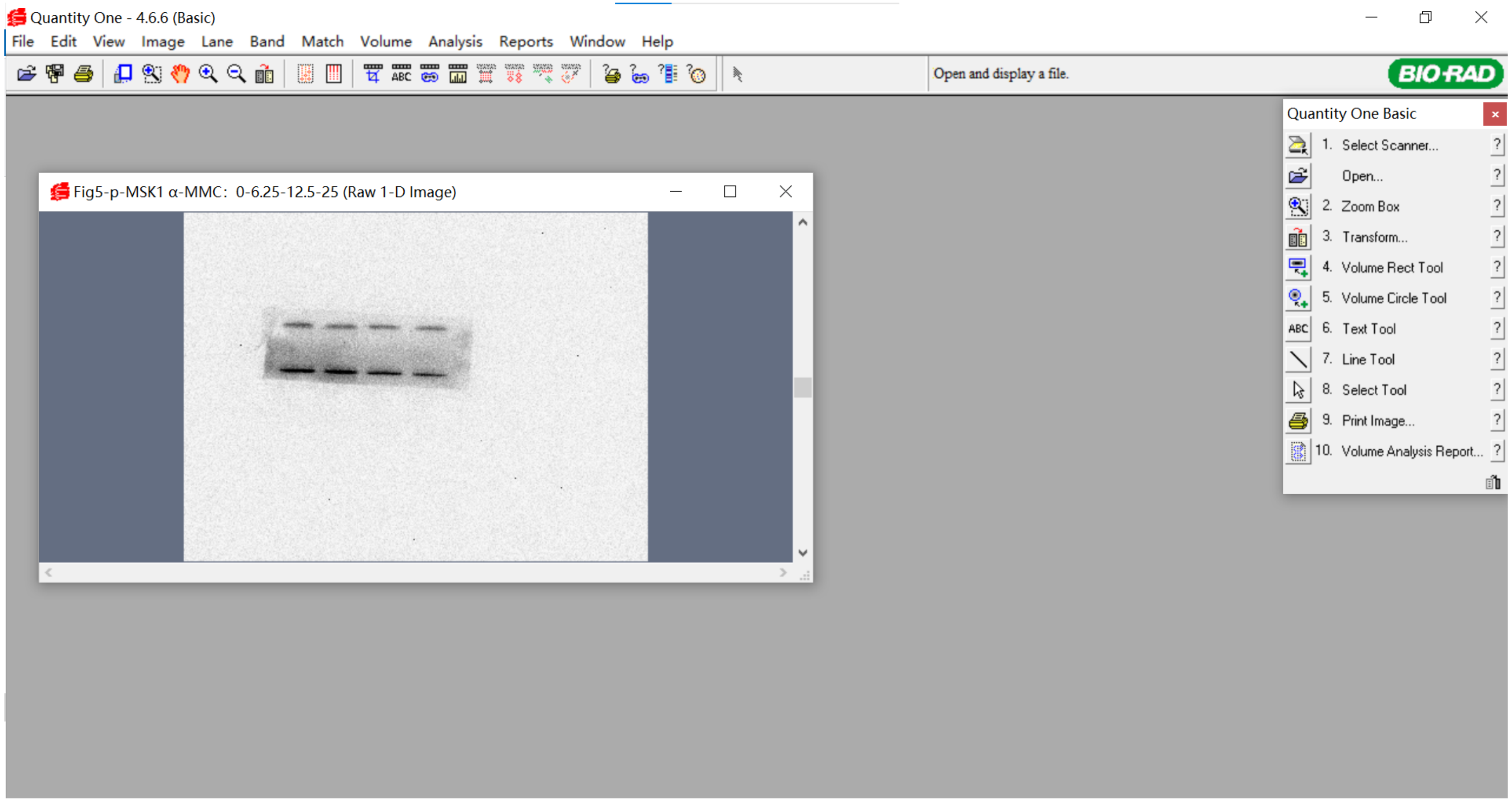

Fig5-p-p38 α-MMC (μg/mL)： 0-6.25-12.5-25

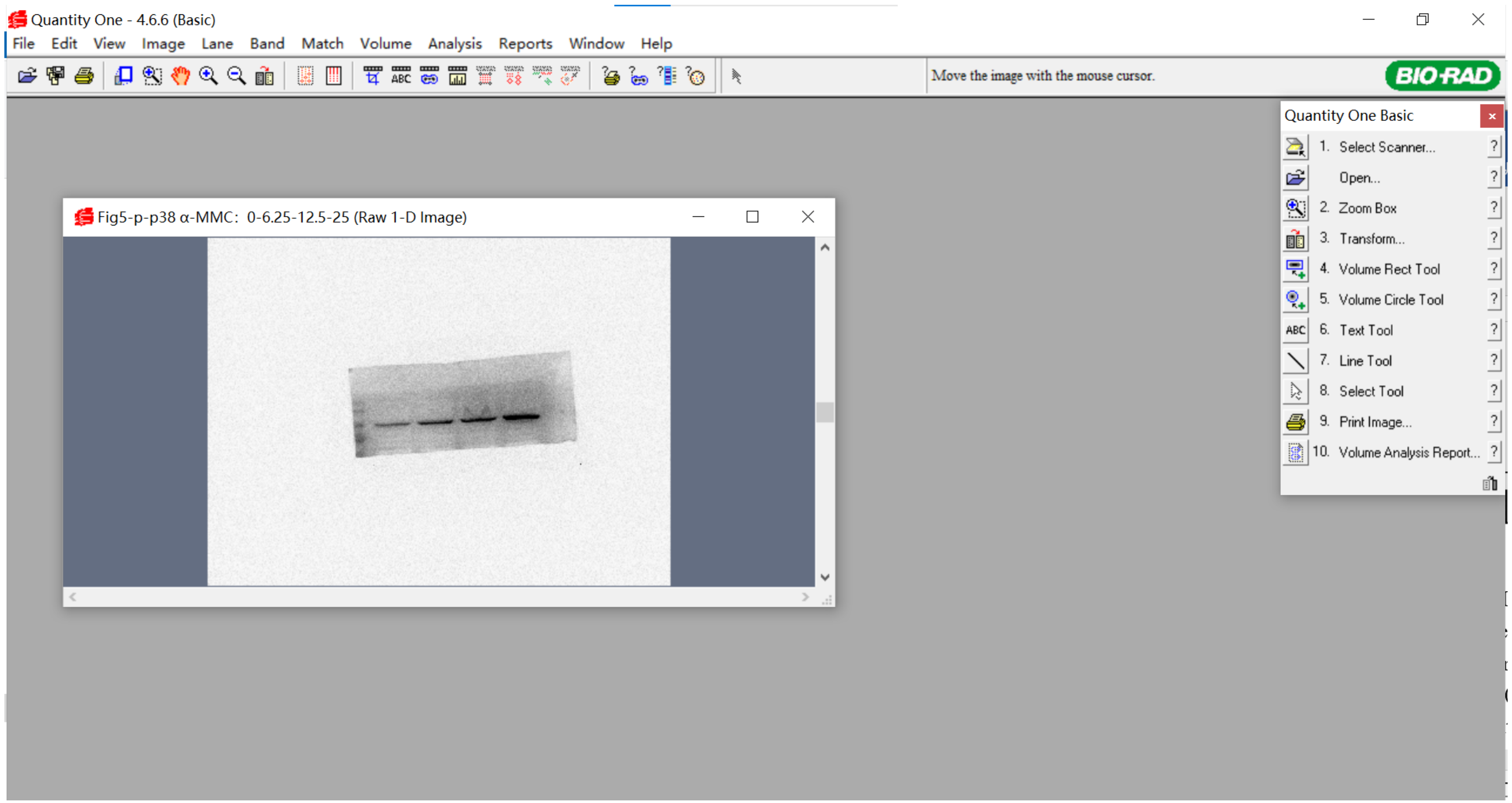

Fig5-p-P90RSK α-MMC (μg/mL)： 0-6.25-12.5-25

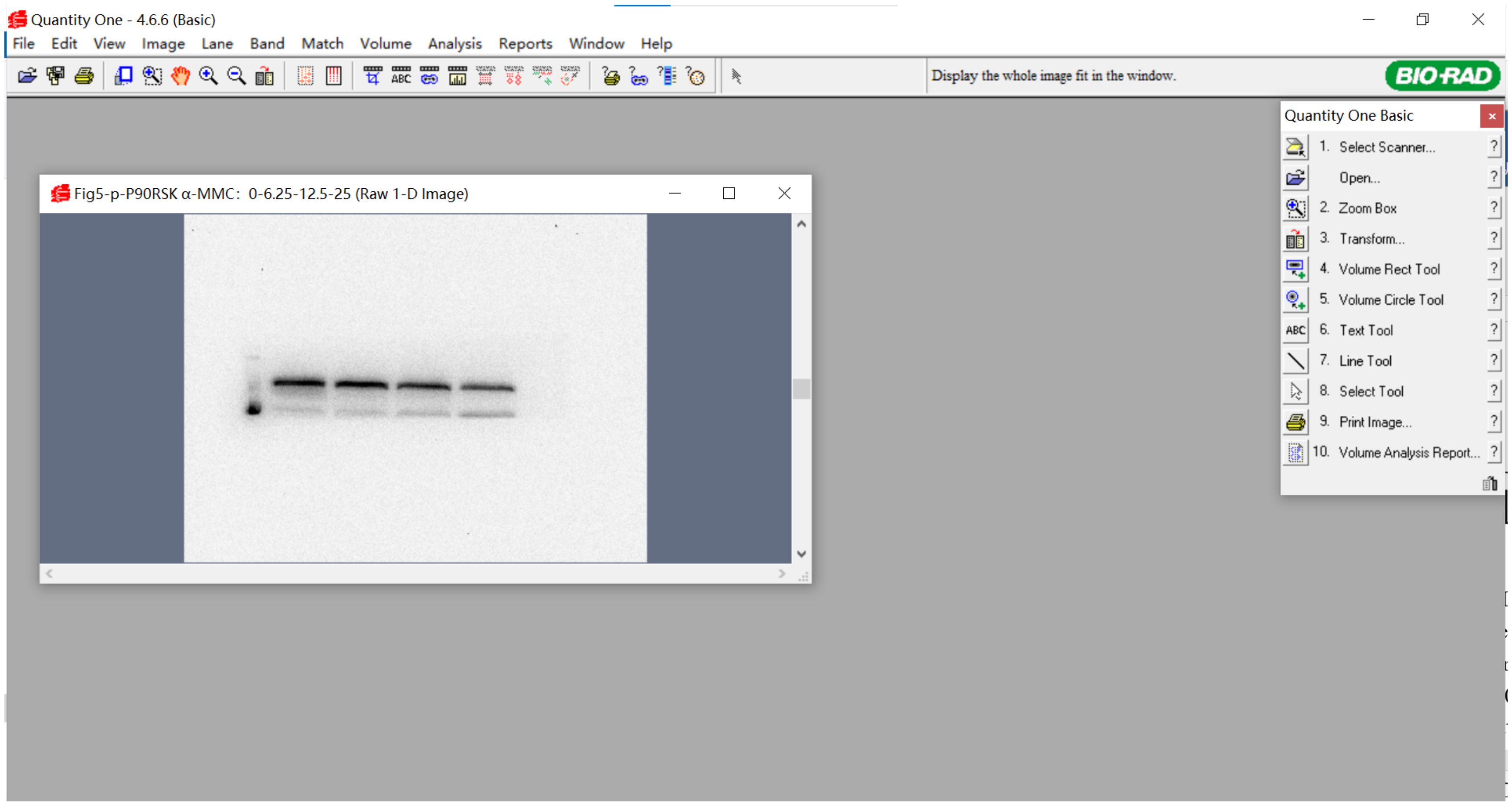

Fig5-p-SPAK_JNK α-MMC (μg/mL)： 0-6.25-12.5-25

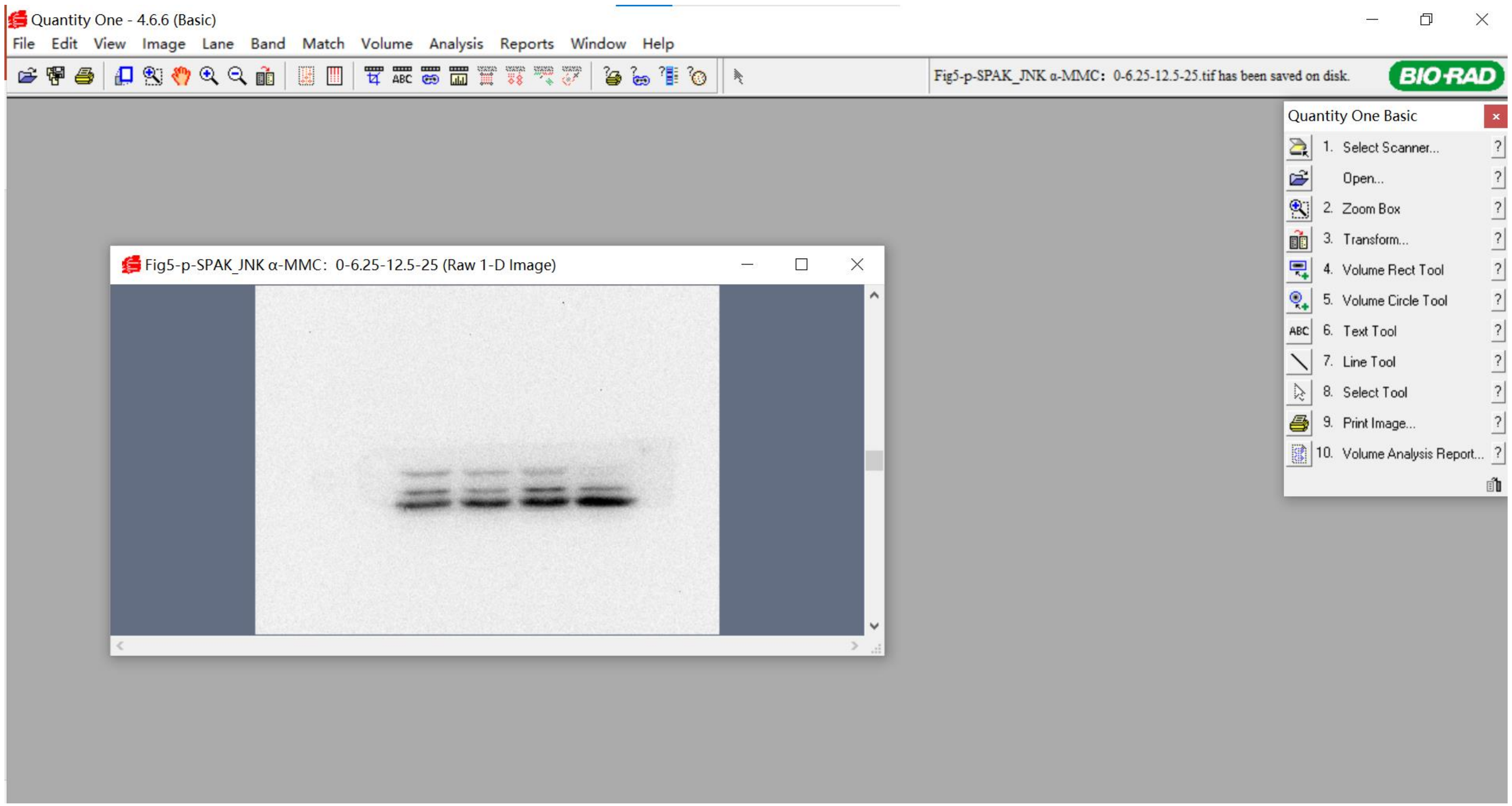

Supplement: Supplementary file 1 [file pharmaceuticals-16-00124-s001.zip › Original Images for Blots.pdf]
