# Supplementary figures and images for: MAPK Cascade Signaling Is Involved in α-MMC Induced Growth Inhibition of Multiple Myeloma MM.1S Cells via G2 Arrest and Mitochondrial-Pathway-Dependent Apoptosis In Vitro
Source: Pharmaceuticals (Basel). 2023 Jan 13;16(1):124. doi: 10.3390/ph16010124 (PMC9867419; doi:10.3390/ph16010124)

0  $\alpha$ -MMC  $\mu\text{g/mL}$

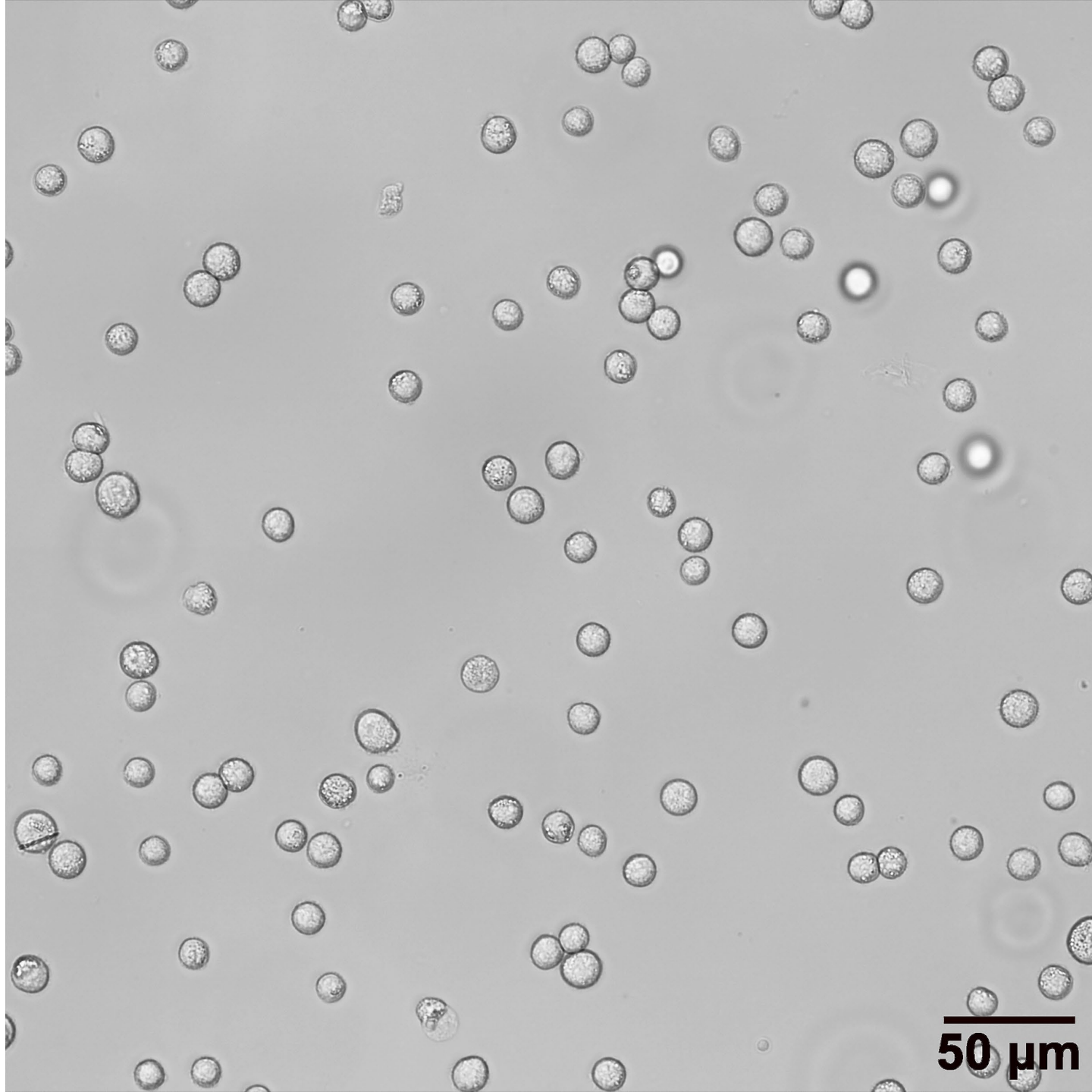

12.5  $\alpha$ -MMC  $\mu\text{g/mL}$

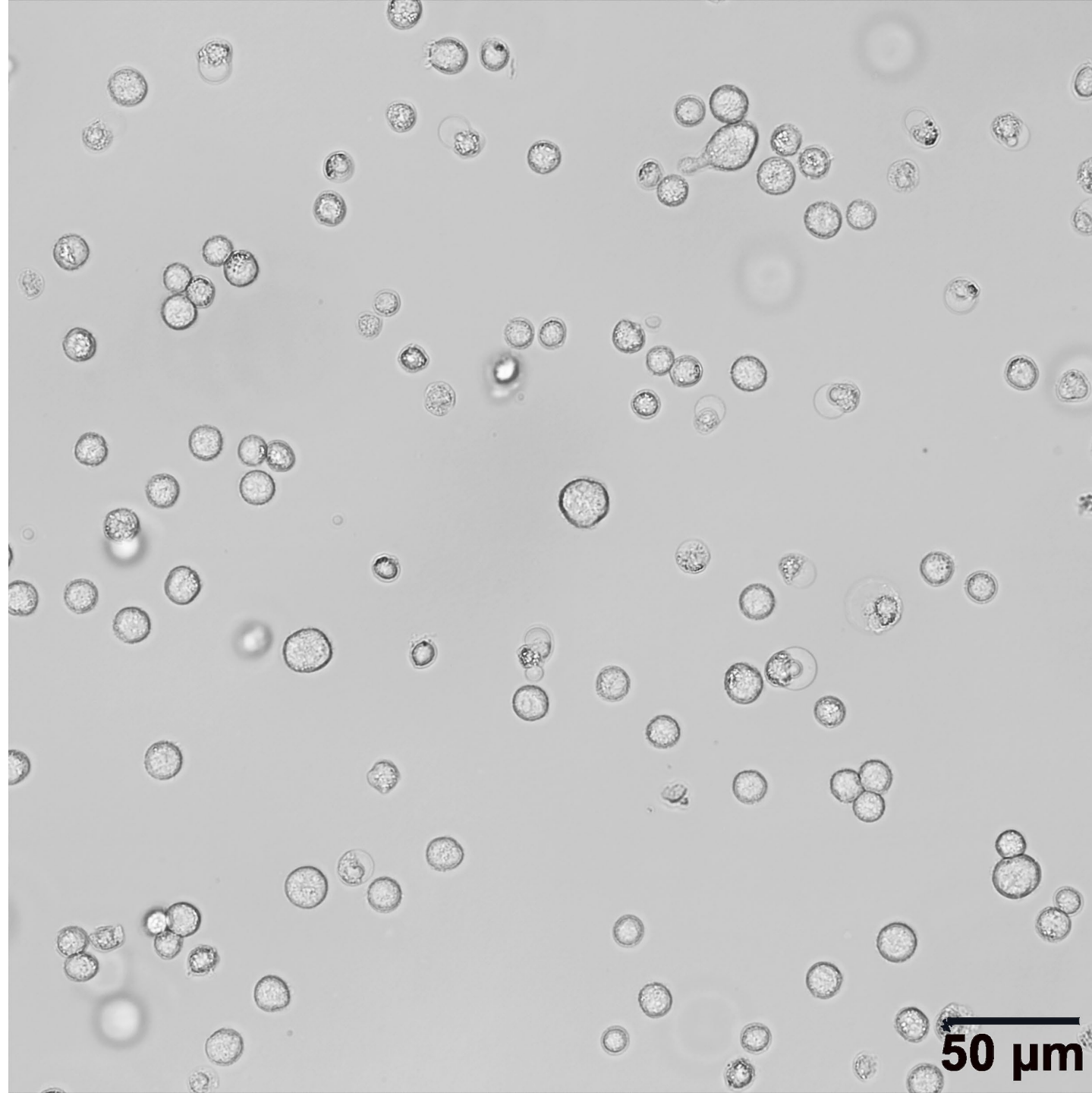

25  $\alpha$ -MMC  $\mu\text{g/mL}$

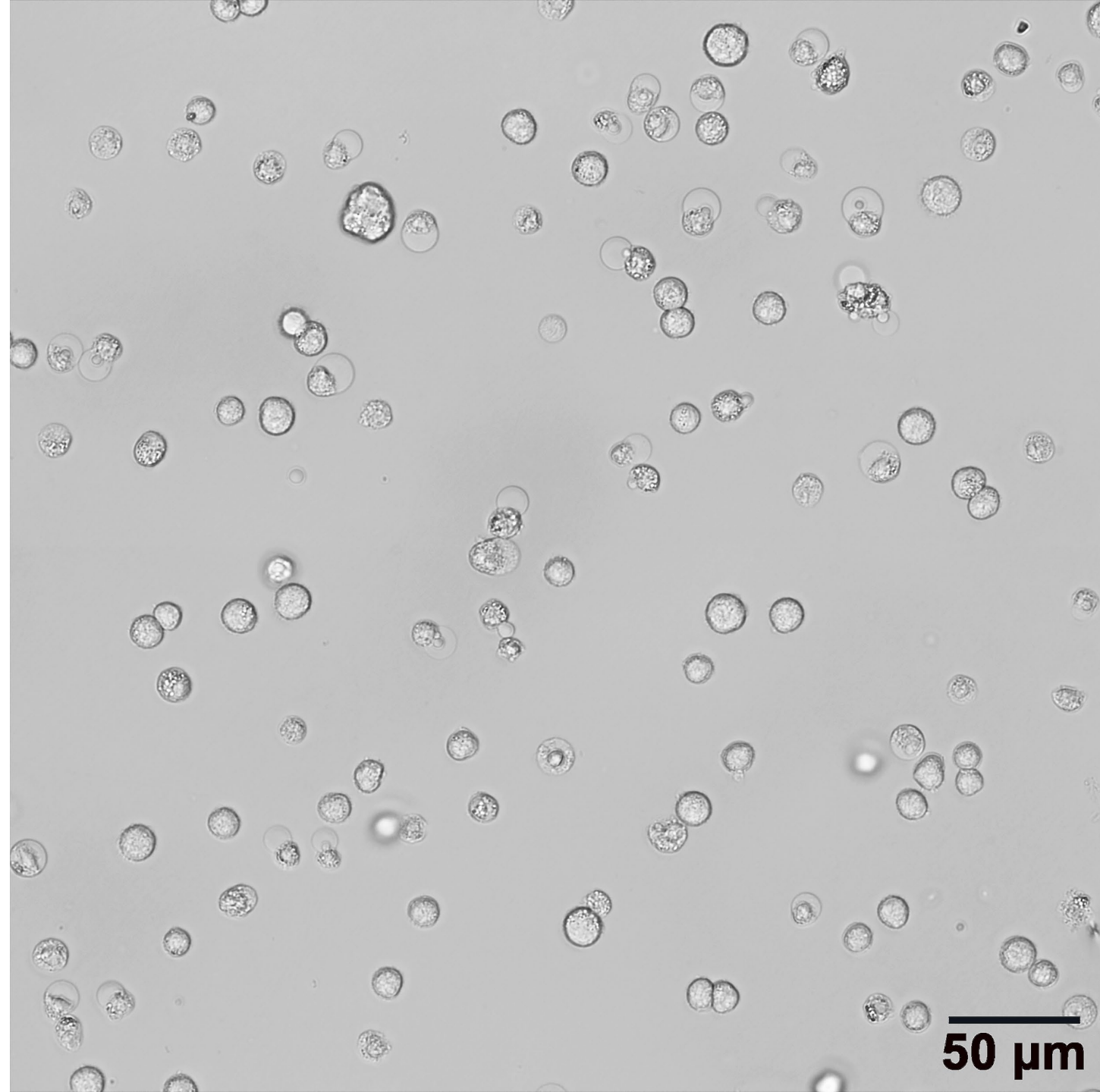

Supplement: Supplementary file 1 [file pharmaceuticals-16-00124-s001.zip › Inverted microscope imaging.pdf]
